# Supplementary material for: Tandem microplastic degradation and hydrogen production by hierarchical carbon nitride-supported single-atom iron catalysts
Source: Nat Commun. 2024 Oct 10;15:8769. doi: 10.1038/s41467-024-53055-1 (PMC11464750; doi:10.1038/s41467-024-53055-1)
Supplement: Supplementary file 1 — Supplementary Information [file 41467_2024_53055_MOESM1_ESM.pdf]

Supplementary Information for

Tandem microplastic degradation and hydrogen production by hierarchical carbon nitride-supported single-atom iron catalysts

Jingkai Lin[1,†], Kunsheng Hu[1,†], Yantao Wang[1], Wenjie Tian[1,2]*, Tony Hall[3], Xiaoguang Duan[1], Hongqi Sun[4], Huayang Zhang[1,2]*, Emiliano Cortés[2] and Shaobin Wang[1]*

[1] School of Chemical Engineering, The University of Adelaide, North Terrace, Adelaide, SA 5005, Australia.

[2] Nanoinstitute Munich, Faculty of Physics, Ludwig-Maximilians-Universität München, Munich, Germany

[3] Mawson Analytical Spectrometry Services, Faculty of Sciences, Engineering and Technology, The University of Adelaide, Adelaide, SA 5005, Australia.

[4] School of Molecular Sciences, The University of Western Australia, Perth, WA 6009, Australia.

Corresponding authors: Wenjie Tian, wenjie.tian@adelaide.edu.au; Huayang Zhang, huayang.zhang@adelaide.edu.au; Shaobin Wang: shaobin.wang@adelaide.edu.au.

This PDF file includes:

Supplementary Discussion
Supplementary Figures 1 to 53
Supplementary Tables 1 to 8
References 1 to 26

Supplementary Discussion

Use of FeSA-hCN with different Fe loadings

The FeSA-hCN catalysts with different Fe loadings (0.5, 2.2 and 4.0 wt%) were synthesized with the variation of Fe precursor amount. The weight percentage of Fe in these catalysts were determined by TGA analysis (Supplementary Fig. 16). Various characterizations were employed to investigate the structural properties of FeSA-hCN catalysts, including XRD (Supplementary Fig. 17), FTIR (Supplementary Fig. 18), XANES (Supplementary Fig. 19), and HAADF-STEM (Supplementary Fig. 20). Specifically, XRD and FTIR spectra suggested that FeSA-hCN can well retain the structure of carbon nitride (Supplementary Figs. 17 and 18). The C K-edge XANES spectra in Supplementary Fig. 18a indicated the π* C–C/C=C interlayer interaction (286 eV) and π* C−N−C (289 eV) of carbon nitride. The N K-edge XANES spectra in Supplementary Fig. 18b showed π* C−N−C (399.5) and π* N−C (402.4 eV) groups of carbon nitride. Noted that the π* C–C/C=C and π* C−N−C groups remained the same after loading single-atom Fe. The intensity of π* N−C groups decreased with the increasing Fe concentration, indicating the loss of π* N−C groups (N–C–2H and 2C–N–H). This demonstrated the variation of C–N groups and the loss of H-bonding interactions between melon chains, confirming the successful incorporation of single-atom Fe between the melon chain of carbon nitride. XRD also indicated the atomic feature of Fe sites in FeSA-hCN catalysts with no metal peak emerged (Supplementary Fig. 17). HAADF-STEM provided direct evidence of the atomic dispersion of Fe throughout the carbon nitride framework (Supplementary Fig. 20). Fe L-edge XANES spectra indicated the valence state of Fe is mainly +2 in FeSA-hCN samples (Supplementary Fig. 19c). In total, the above results confirm the successful synthesis of FeSA-hCN with varying Fe loading contents. Considering the same XRD, FTIR, and XAS results, it is speculated that FeSA-hCN catalysts own the same structural properties but different atomic Fe site densities.

Supplementary Figures

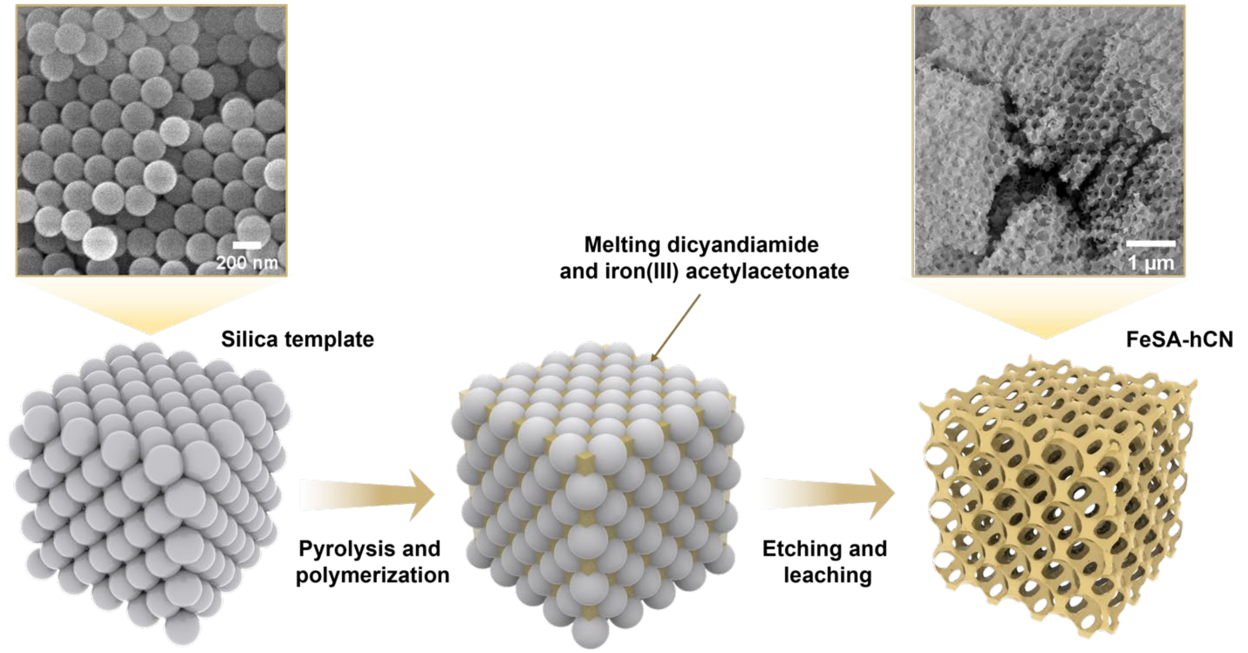

Supplementary Fig. 1 | Synthesis procedure. Schematic illustration of synthesis procedure of FeSA-hCN.

The corresponding SEM images of the silica template and FeSA-hCN were provided. FeSA-hCN was synthesized using a silica hard-template confined pyrolysis method. Firstly, an ordered silica template with diameters of about 250 nm was synthesized via a modified Stöber method[1]. Well-mixed dicyandiamide (DCD) and iron (III) acetylacetonate ($Fe(acac)_3$) precursors were uniformly tiled on the surface of the silica templates, which will melt and infiltrate into the void between adjacent silica spheres during the stepwise heat treatment under nitrogen at 520 °C for 2 h and 550 °C for 2 h. The gaps between adjacent silica spheres provide a uniform spatial confinement effect on the precursors, facilitating the immobilization of single-atom Fe on CN framework. After subsequent etching and template removal, FeSA-hCN SACs were produced.

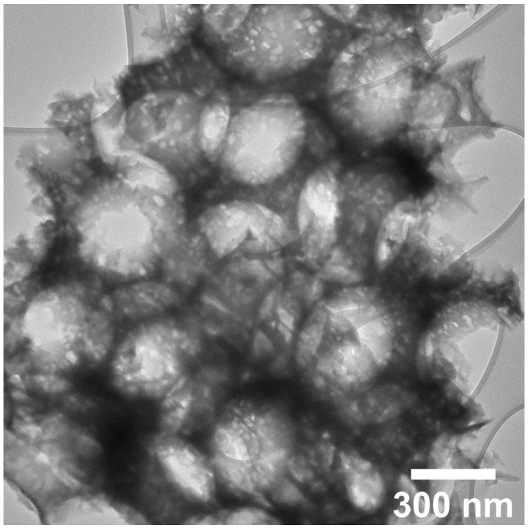

Supplementary Fig. 2 | Porous structure characterization. TEM image of FeSA-hCN.

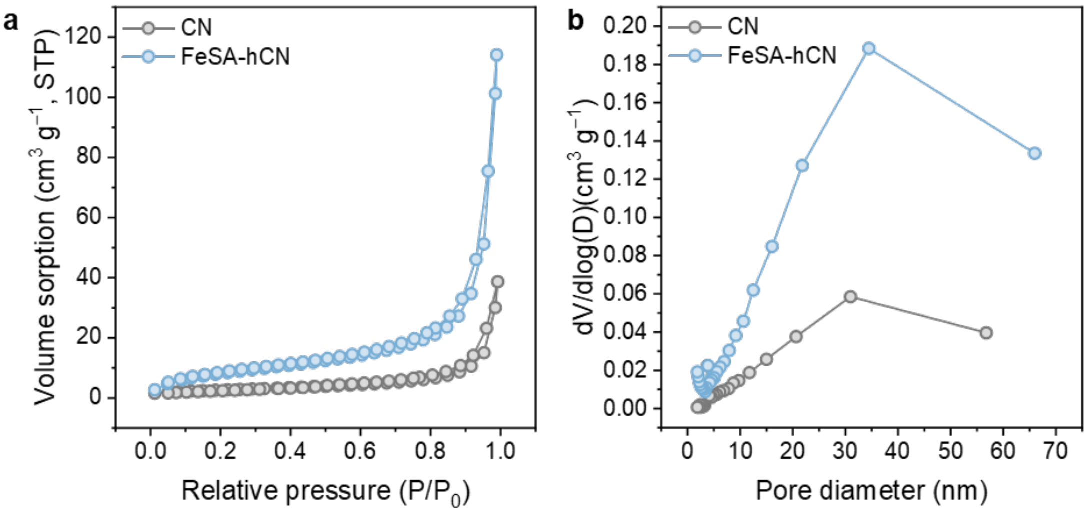

Supplementary Fig. 3 | Surface properties analysis. a, BET analysis of nitrogen adsorption-desorption isotherm. **b**, pore-size distribution curves measured by the BJH distribution method.

As shown in Supplementary Fig. 3a above, N_2 sorption isotherms revealed FeSA-hCN owned a high Brunauer–Emmett–Teller surface area of 31.4 m^2 g^{-1} and a total pore volume of 0.18 cm^3 g^{-1} (Supplementary Fig. 3 and Table 3). Barret-Joyner-Halender (BJH) pore size in Supplementary Fig. 3b distribution curves also revealed abundant mesopores in the macroporous structure of FeSA-hCN. This agrees well with the SEM and TEM results.

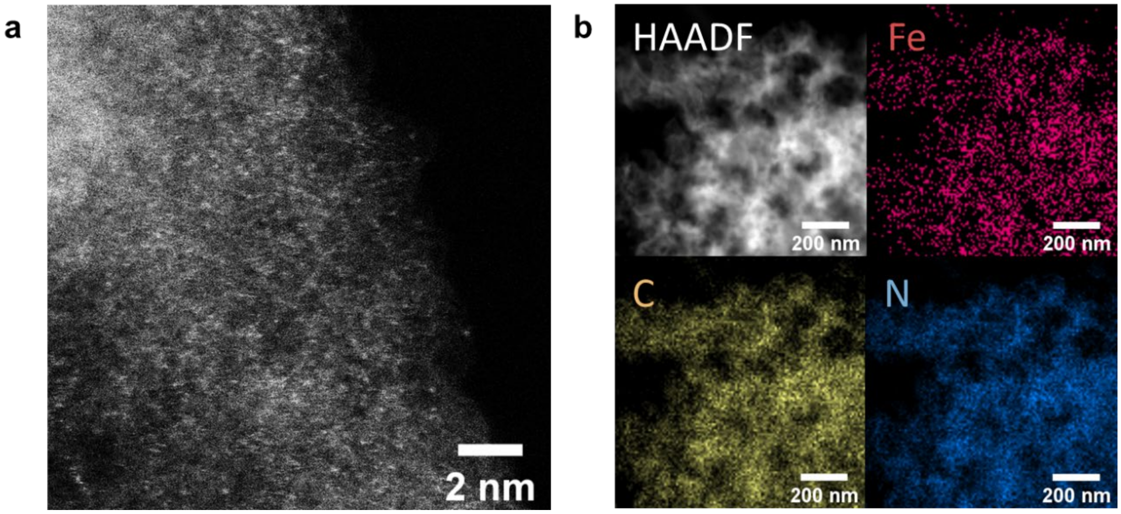

Supplementary Fig. 4 | High-angle annular dark field scanning transmission electron microscopy (HAADF-STEM) characterizations of FeSA-hCN. a, Aberration-corrected (AC) HAADF-STEM image. **b**, HAADF-STEM image and corresponding elemental mappings.

HAADF-STEM indicates the atomic dispersion of Fe species on FeSA-hCN. Energy-dispersed X-ray spectroscopy (EDX) elemental mappings further confirmed the uniform distribution of Fe, N, and C elements throughout the FeSA-hCN structure.

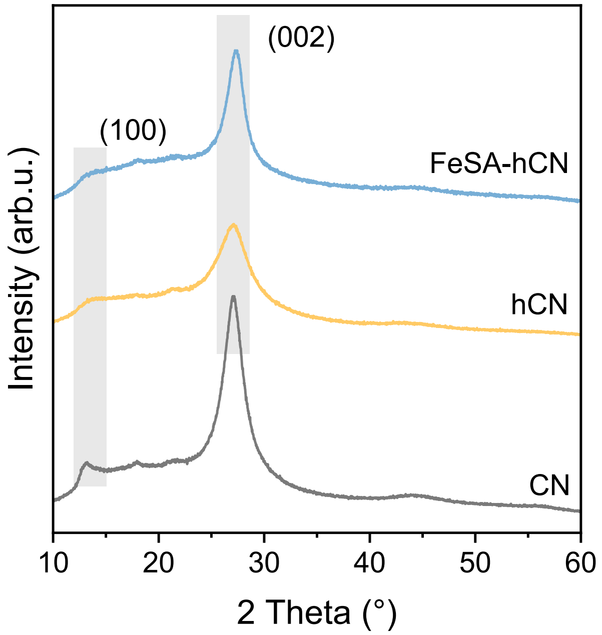

Supplementary Fig. 5 | X-ray diffraction patterns (XRD) analysis. XRD of FeSA-hCN, hCN and CN.

In XRD patterns, there are two characteristic peaks at around 13° and 27°, which correspond to the (100) and (002) planes of carbon nitride[2]. The characteristic peak (100) was distributed to the in-plane ordering of the tri-s-triazine units. The sharp characteristic peak (002) near 27° was assigned to the interlayer stacking of the conjugated aromatic systems. Noted that the intensity of the (002) peak decreased after engineering macroporous structure on carbon nitride. This may be due to the formation of macrouporous structure, which will inhibit the interlayer periodicity stacking of carbon nitride and reduce the interlayer stacking of the conjugated aromatic segments[3,4]. Loading single-atom Fe on hCN allows the coupling conjunction between Fe and the Lewis base N atoms in the carbon nitride, increasing the crystallization through the arrangement of the conjugated heptazine units[2,5], thus, increasing the intensity of the (001) and (002) peaks. This confirmed the successful loading of single-atom Fe in the carbon nitride framework.

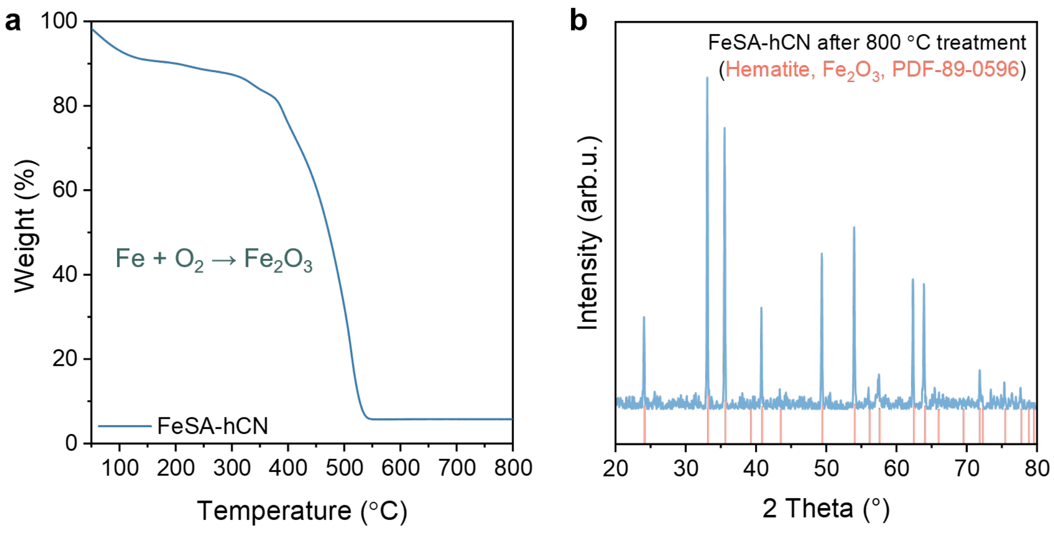

Supplementary Fig. 6 | Fe weight percentage analysis. **a**, TGA analysis of FeSA-hCN. **b**, XRD analysis of the FeSA-hCN after 800 °C treatment (air).

The quantification of metal content in FeSA-hCN was conducted through TGA. The sample underwent pyrolysis up to 800 °C in an airflow environment, leading to a weight loss attributed to the removal of H, C, N, and O. The utilization of air as the purging gas caused the oxidation of Fe into Fe_2O_3. This was confirmed by XRD analysis. The remaining weight corresponded to the Fe_2O_3 content. Considering the Fe percentage within Fe_2O_3, we calculated the initial Fe weight percentage in FeSA-hCN to be 4.0 wt%.

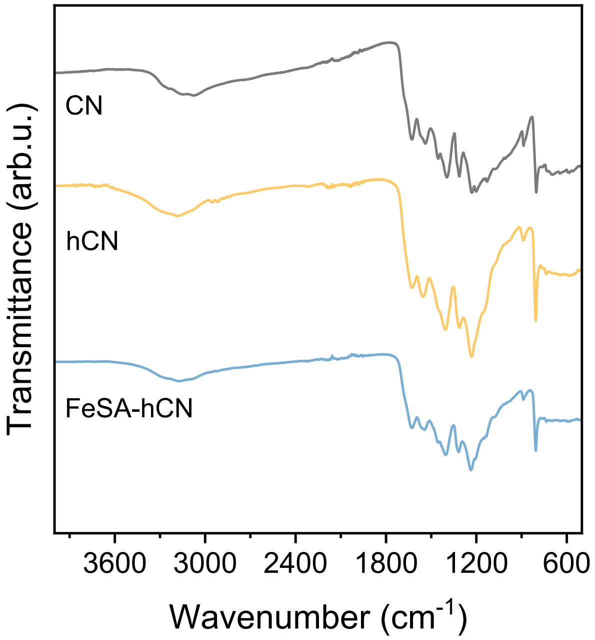

Supplementary Fig. 7 | Fourier transform infrared spectroscopy (FTIR) analysis. FTIR spectra of FeSA-hCN, hCN and CN.

The thermal condensation of DCD at 550 °C promotes the formation of hydrogen-bonded polymeric melon structures, maximizing the presence of NH/NH_2 groups within the material[6]. The Fourier transform infrared spectroscopy (FTIR) spectrum of the as-prepared FeSA-hCN closely resembles the vibrational spectra found in the literature for melon-structured carbon nitride. Specifically, the band at 810 cm^{-1} is attributed to the ring sextant out-of-plane bending vibration. Bands within the range of 1250 to 1650 cm^{-1} correspond to C−N heterocycles in carbon nitride. The presence of NH/NH_2 groups, integral components of the structure, is confirmed by the N−H stretching region observed between 3250 and 3070 cm^{-1}. These findings indicate the presence of a hydrogen-bonded polymeric melon structure in carbon nitride, featuring NH/NH_2 groups, which provides an ideal framework for anchoring Fe single atoms (Supplementary Fig. 8).

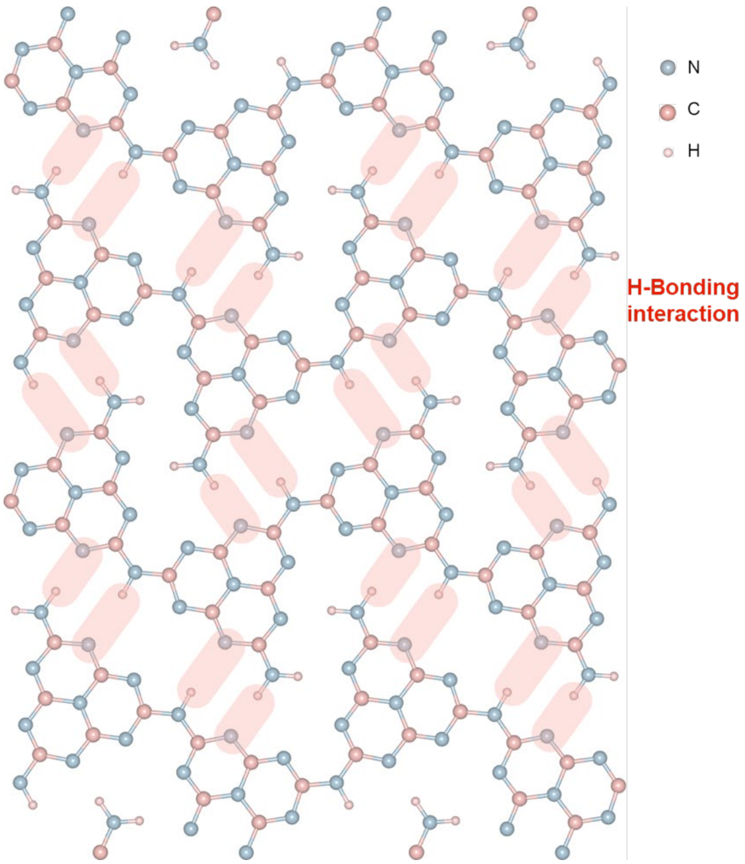

Supplementary Fig. 8 | Structure illustration. Illustration of atomic structure of hydrogen-bonded polymeric melon-based carbon nitride.

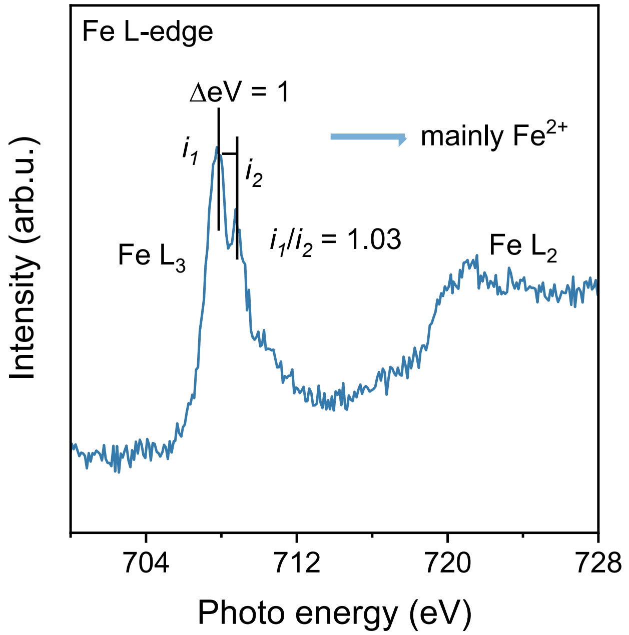

Supplementary Fig. 9 | XANES spectra. Fe $L_{2,3}$-edge XANES spectra of FeSA-hCN.

The Fe L-edge XANES spectrea showed two typical L_3 and L_2 peaks at about 708 and 721 eV, respectively, which were attributed to the Fe electronic transitions from 2*p* orbitals to the unoccupied 3*d* orbitals [7]. The valence states of Fe in FeSA-hCN were explored by analyzing the intensity ratio (absorption intensity i_1/i_2) and the energy difference (ΔeV) between peaks i_1 and i_2 in Fe L-edge XANES spectra. The calculated ΔeV of ~1 and intensity ratio of ~1.03 suggest that Fe species mainly exist as Fe^{2+} in FeSA-hCN (Supplementary Table 4)[8].

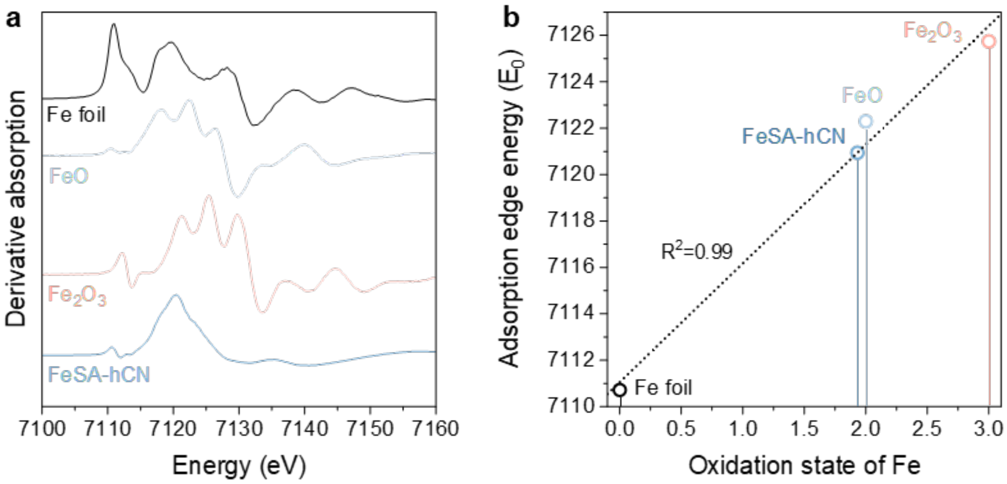

Supplementary Fig. 10 | Oxidation state analysis. First derivatives of Fe K-edge XANES and oxidation states estimation.

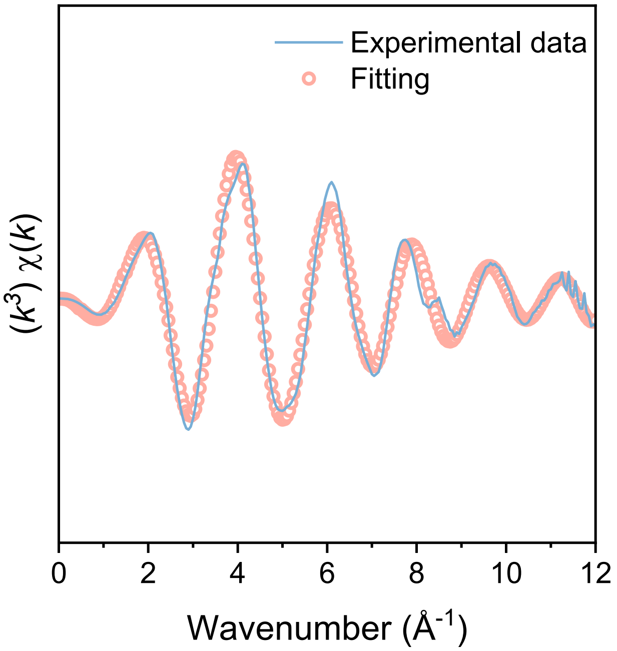

Supplementary Fig. 11 | Best EXAFS fitting. Fitted EXAFS in K-space of FeSA-hCN.

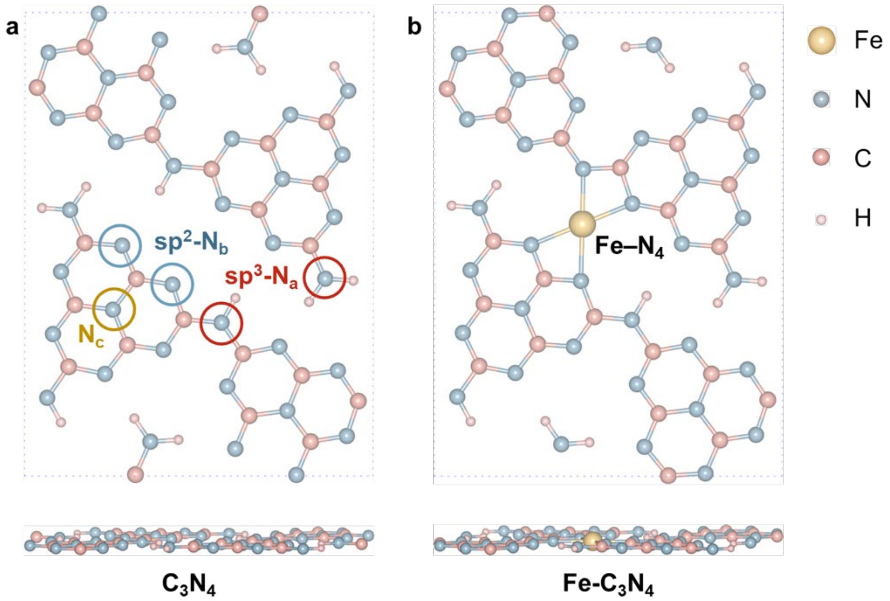

Supplementary Fig. 12 | Simulated models for theoretical calculation. a, C_3N_4 and **b**, Fe-C_3N_4 for the representation of CN and FeSA-hCN.

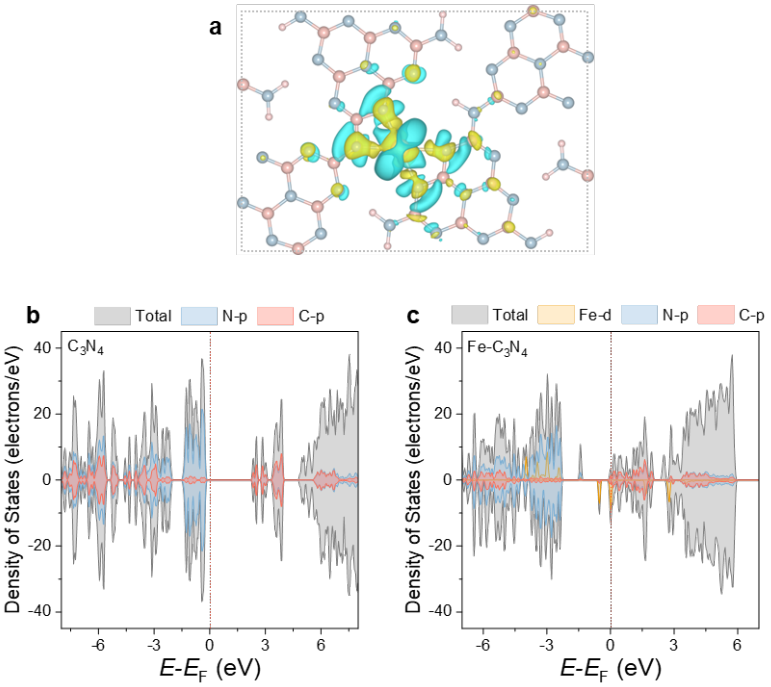

Supplementary Fig. 13 | Electronic structure analysis using DFT simulation. a, The differential charge density of Fe-C_3N_4. Color legend for isosurface: cyan, charge depletion; yellow, charge accumulation. **b and c,** Total and projected density of states (DOS) of C_3N_4 and Fe-C_3N_4.

According to the optimized theoretical model, the variation of the electron density was studied using density functional theory (DFT) calculations. The presence of Fe–N_4 sites led to a charge redistribution[9,10] (Supplementary Fig. 13a). As observed from the total and projected density of state (DOS), the carbon nitride possessed a semiconducting feature with relatively poor electronic conductivity (Supplementary Fig. 13b). In comparison, the introduction of single-atom Fe sites resulted in the display of prominent electronic states of N 2*p*, C 2*p*, and Fe 3*d* near the Fermi level (Supplementary Fig. 13c). This was attributed to the charge redistribution among C and N atoms in carbon nitride, which could alter the electronic structure and improve the conductivity[2,9]. Notably, the hybridization of the Fe 3*d* and N 2*p* orbitals near the Fermi level indicated the formation of the strong Fe–N covalent bond, which prominently enhanced the charge transfer. Theoretical analysis verified that the introduction and interaction of single-atom Fe sites within the carbon nitride framework could modulate the electronic structure of carbon nitride through charge redistribution, which would affect the catalytic performance of the material.

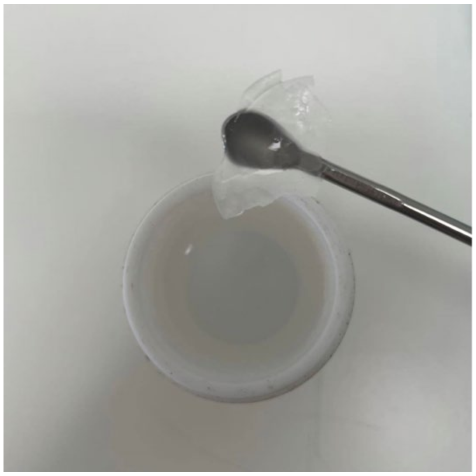

Supplementary Fig. 14 | Reaction solution in a bare H_2O_2 system after hydrothermal UHMWPE MPs degradation test. UHMWPE powders in a bare H_2O_2 system after hydrothermal treatment aggregated into a large piece. Only 31.4 wt% MPs weight loss was achieved. Reaction conditions: [UHMWPE MPs] = 1 g L^{-1}, Catalyst was not added, [H_2O_2] = 100 mM, hydrothermal temperature = 140 °C and neutral pH.

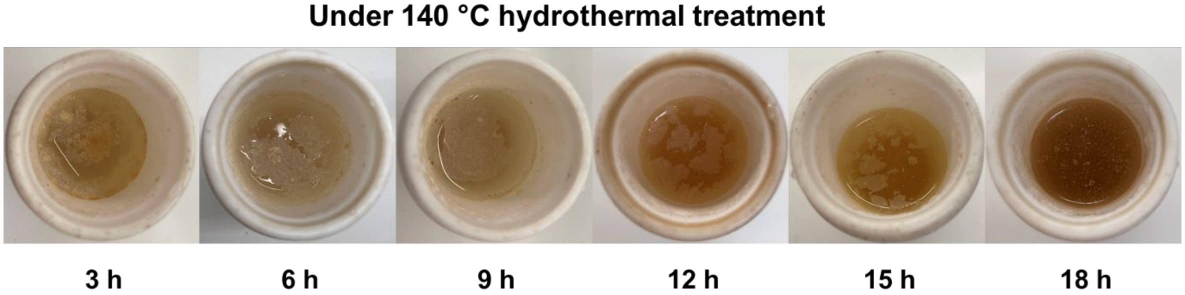

Supplementary Fig. 15 | Reaction solution of FeSA-hCN/H_2O_2 systems after hydrothermal UHMWPE degradation. Reaction conditions: [UHMWPE MPs] = 1 g L^{-1}, [catalysts] = 1 g L^{-1}, [H_2O_2] = 100 mM, hydrothermal temperature = 140 °C, and neutral pH.

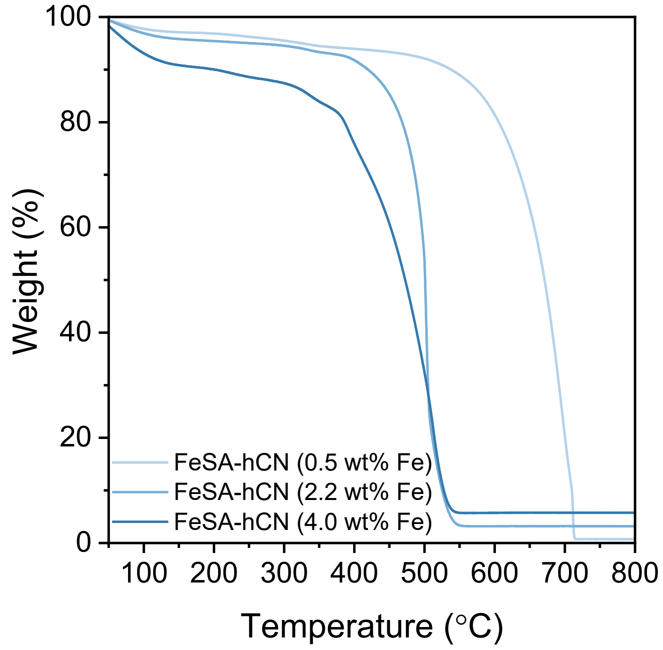

Supplementary Fig. 16 | Fe weight percentage analysis. TGA of FeSA-hCN with different Fe loadings (air flow).

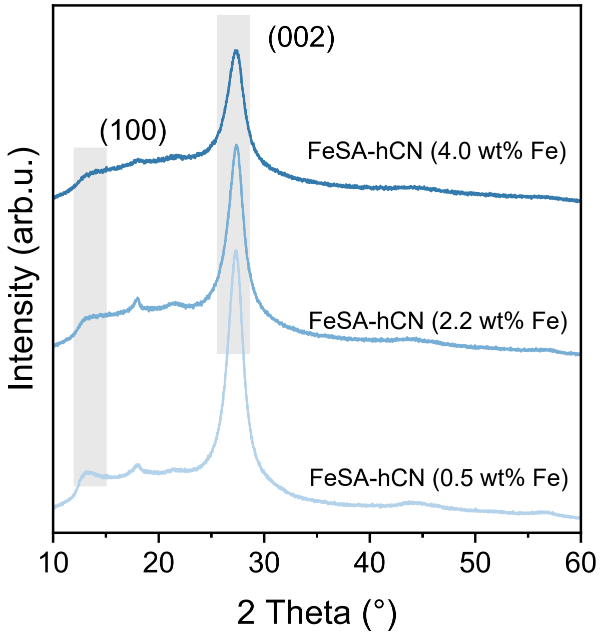

Supplementary Fig. 17 | XRD analysis. XRD of FeSA-hCN with different Fe loadings.

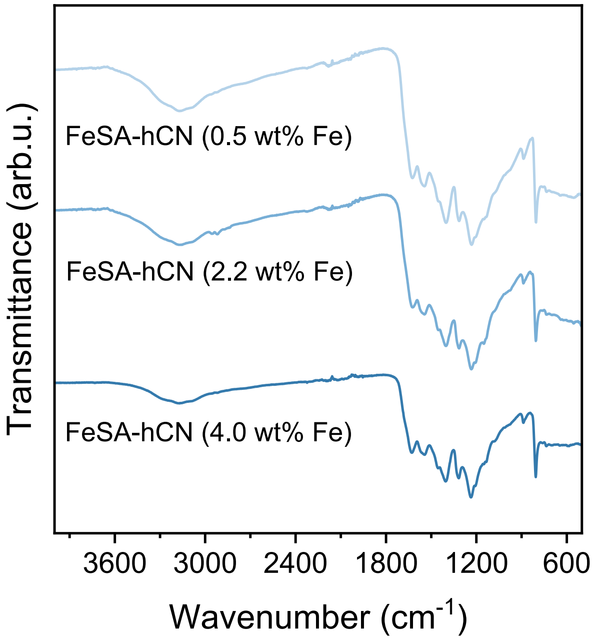

Supplementary Fig. 18 | FTIR analysis. FTIR of FeSA-hCN with different Fe loadings.

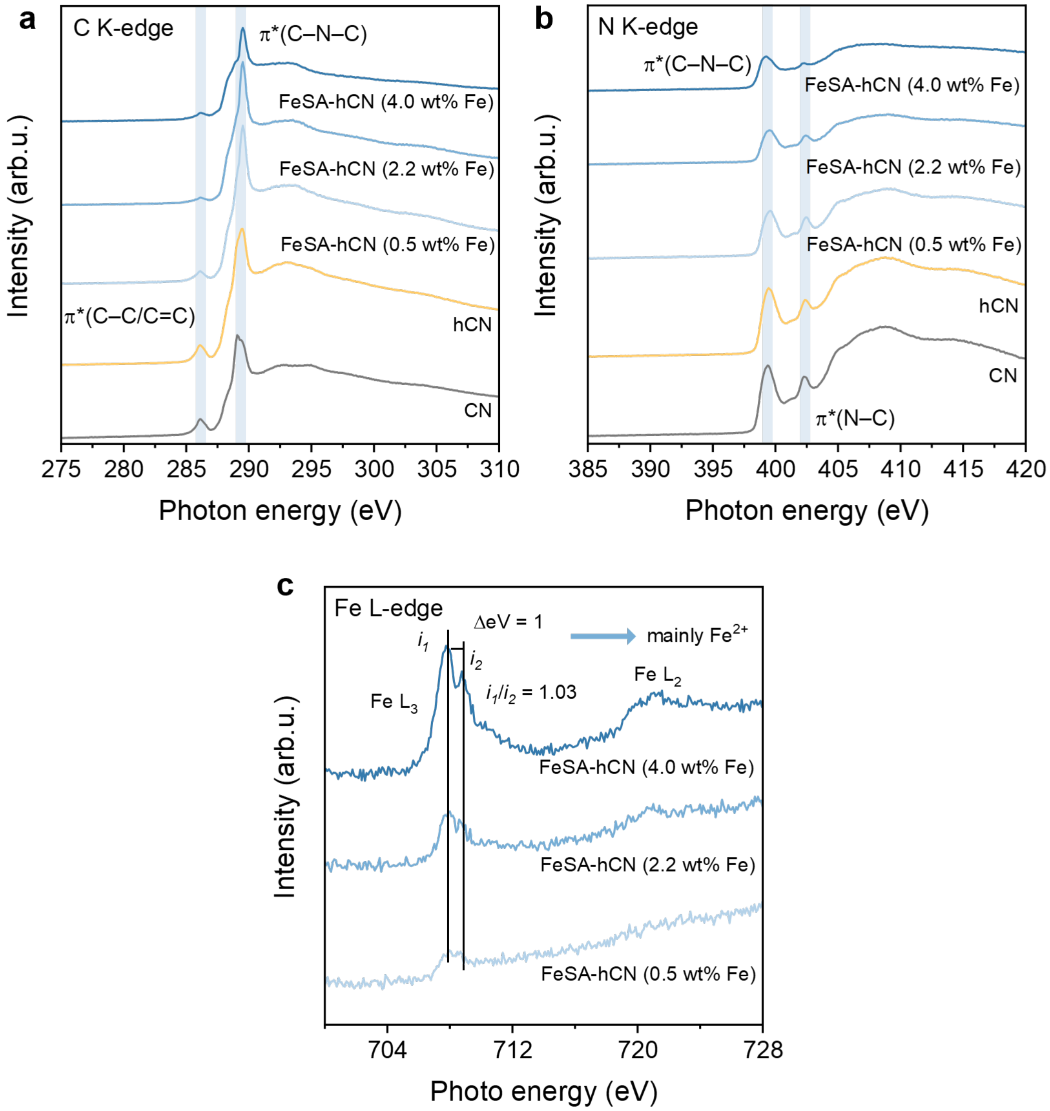

Supplementary Fig. 19 | XANES analysis of FeSA-hCN with different Fe loadings. a, C K-edge XANES analysis. **b,** N K-edge XANES analysis. **c**, Fe L-edge XANES analysis.

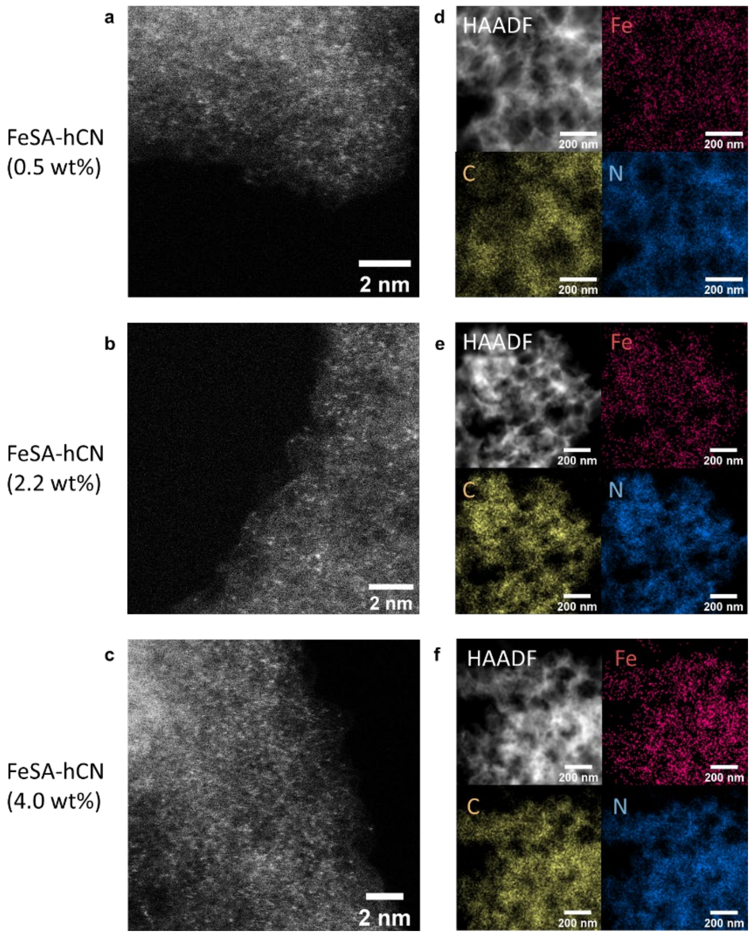

Supplementary Fig. 20 | HAADF-STEM characterizations. a-c, AC HAADF-STEM images and **d-f**, corresponding HAADF-STEM images and elemental mappings of FeSA-hCN with different Fe loadings, ranging from 0.5, 2.2 and 4.0 wt%.

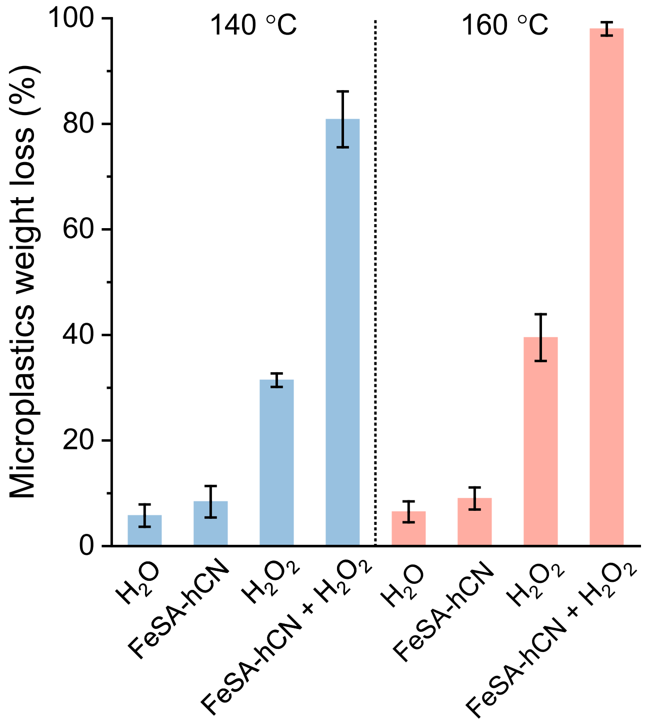

Supplementary Fig. 21 | Degradation performances comparison. UHMWPE MPs degradation performances in different control systems. Reaction conditions: [UHMWPE MPs] = 1 g L^{-1}, [catalyst] = 1 g L^{-1} if used, [H_2O_2] = 100 mM if used, hydrothermal temperature = 140 or 160 °C, reaction time of 12 h and neutral pH. The error bars represent the standard deviations from triplicate tests.

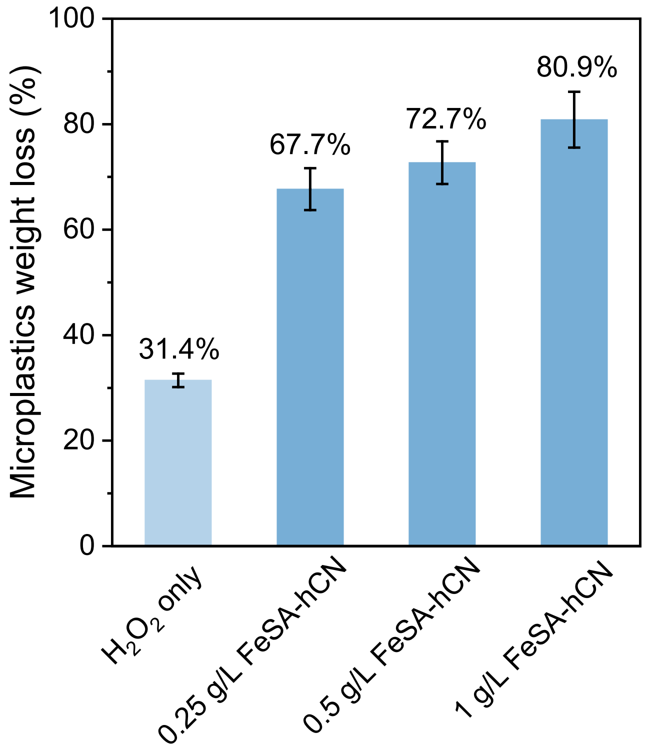

Supplementary Fig. 22 | Effect of FeSA-hCN dosage. Change of FeSA-hCN dosage in the hydrothermal Fenton-like system for UHMWPE MPs degradation. Reaction conditions: [UHMWPE MPs] = 1 g L^{-1}, [catalysts] = 1 g L^{-1}, [H_2O_2] = 100 mM, hydrothermal temperature = 140 °C and neutral pH. The error bars represent the standard deviations from triplicate tests.

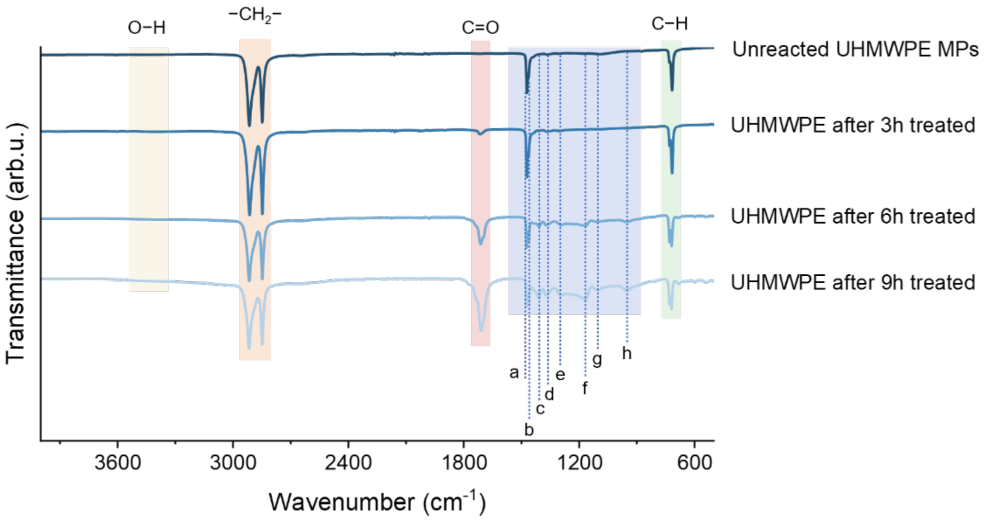

Notes:
a. In-plane CH_2 bending vibration
b. In-plane CH_3 asymmetric bending
c. CH_2 group adjacent to the carbonyl group in ketones
d. CH_3 asymmetric deformation
e. CH_2 out of plane vibration
f. C−O stretching
g. C−O−C
h. O−H out of plane bending vibration in COOH

Supplementary Fig. 23 | FTIR analysis. FTIR of UHMWPE MPs separated and collected after reactions at different time in FeSA-hCN/H_2O_2.

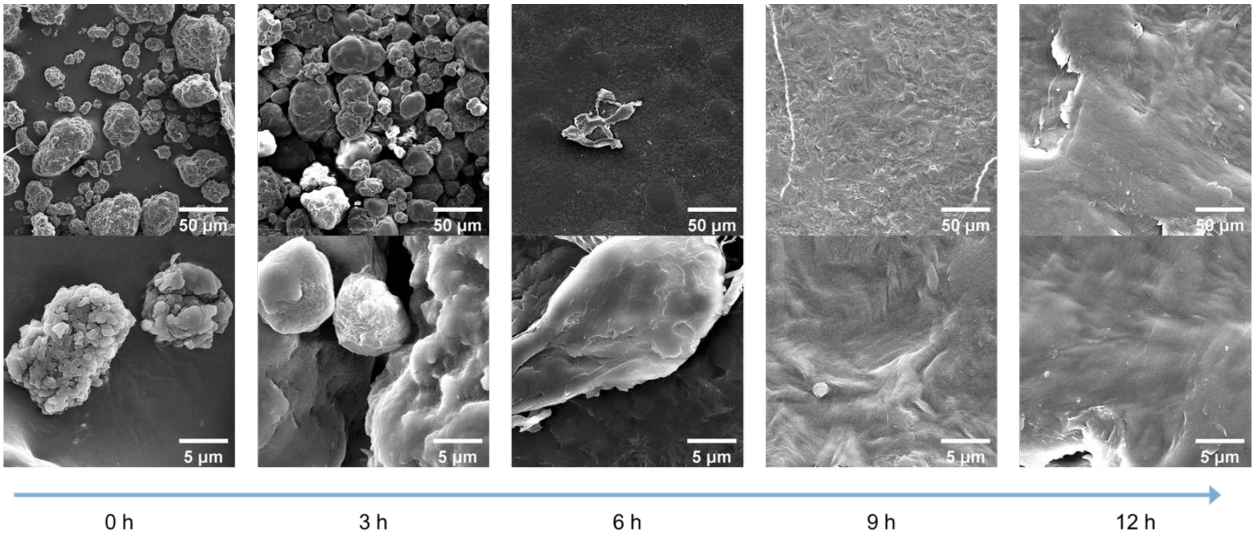

Supplementary Fig. 24 | SEM characterization. SEM images of UHMWPE MPs collected after reaction in a pure H_2O system at 140 °C. Reaction conditions: [UHMWPE MPs] = 1 g L^{-1}, hydrothermal temperature = 140 °C and neutral pH.

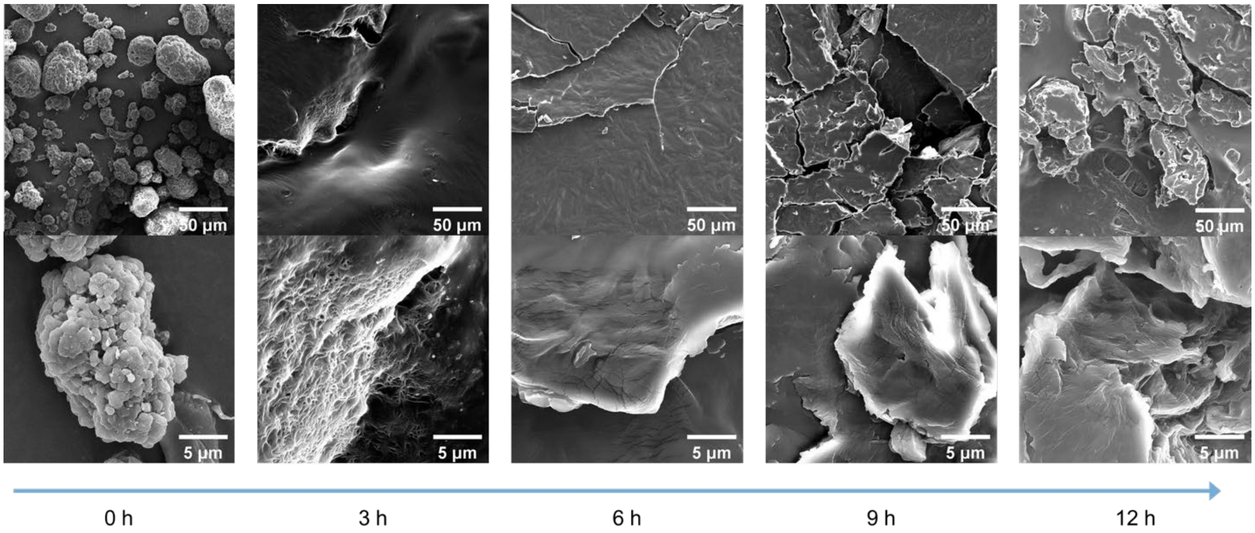

Supplementary Fig. 25 | SEM characterization. SEM images of UHMWPE MPs collected after reaction in a control H_2O_2-only system at 140 °C. Reaction conditions: [UHMWPE MPs] = 1 g L^{-1}, [H_2O_2] = 100 mM, hydrothermal temperature = 140 °C and neutral pH.

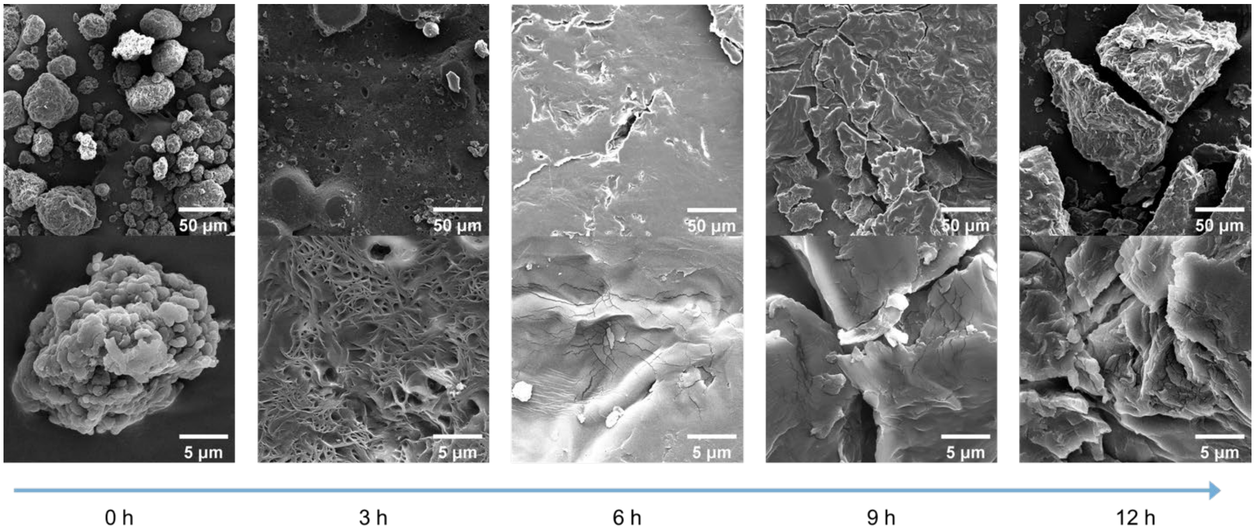

Supplementary Fig. 26 | SEM characterization. SEM images of UHMWPE MPs collected after reaction in a control CN/H_2O_2 system at 140 °C. Reaction conditions: [UHMWPE MPs] = 1 g L^{-1}, [CN] = 1 g L^{-1}, [H_2O_2] = 100 mM, hydrothermal temperature = 140 °C and neutral pH.

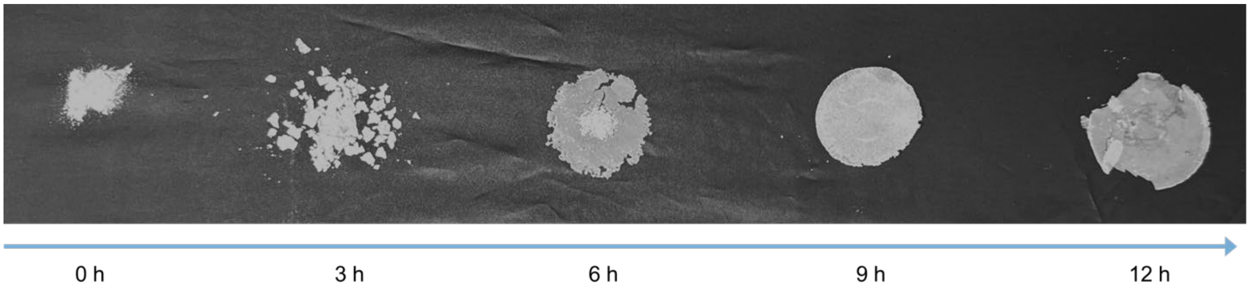

Supplementary Fig. 27 | Plastics after reaction. Photos of UHMWPE MPs after reaction in a pure H_2O system at 140 °C. Reaction conditions: [UHMWPE MPs] = 1 g L^{-1}, hydrothermal temperature = 140 °C and neutral pH.

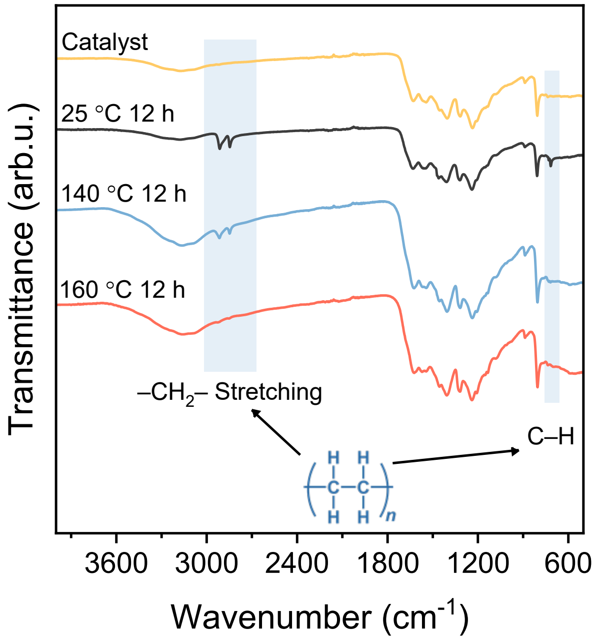

Supplementary Fig. 28 | FTIR analysis of reaction residues. The reaction residues contained FeSA-hCN and unreacted UHMWPE MPs after hydrothermal Fenton-like UHMWPE MPs degradation. Reaction conditions: [UHMWPE MPs] = 1 g L^{-1}, [catalysts] = 1 g L^{-1}, [H_2O_2] = 100 mM, hydrothermal temperature = 140 °C and neutral pH.

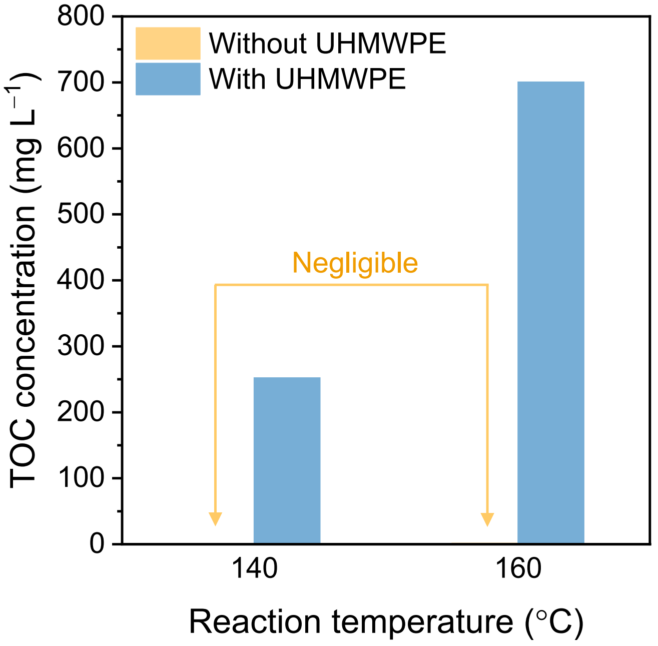

Supplementary Fig. 29 | TOC analysis of reaction solution. TOC concentration of the reaction solution after hydrothermal treatment. Reaction conditions: [UHMWPE MPs] = 1 g L^{-1} if present, [catalysts] = 1 g L^{-1}, [H_2O_2] = 100 mM, hydrothermal temperature = 140 or 160 °C and neutral pH.

A controlled experiment with only FeSA-hCN and H_2O_2 shows that negligible TOC was detected, confirming that all organic carbon species derived from UHMWPE degradation. Noted that a small amount of inorganic carbon (IC) ascribing to the decomposition of FeSA-hCN was detected in the solution, which was much lower than the original carbon proportion in the catalyst, indicating a negligible weight loss of the catalyst sample and confirming its structural stability under hydrothermal conditions.

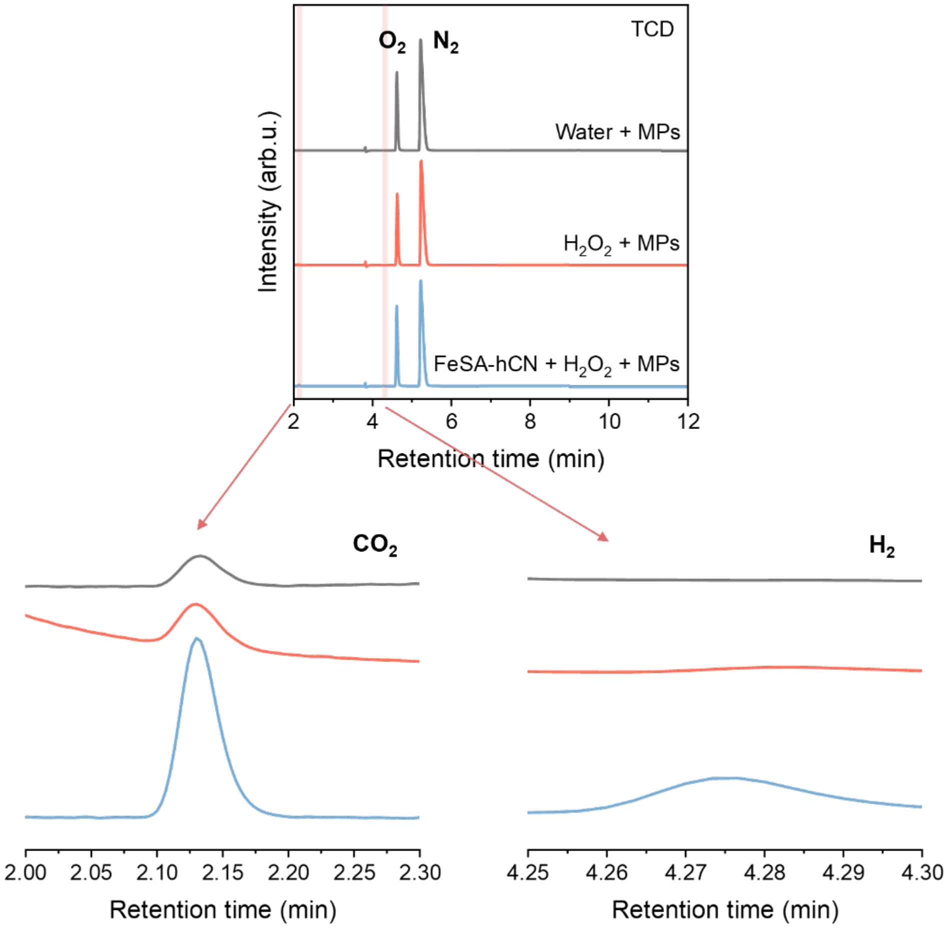

Supplementary Fig. 30 | Gas production during reaction. Detection of gases produced after the hydrothermal UHMWPE MPs degradation using the 8890 GC-TCD detector. Reaction conditions: reaction time = 18 h, [UHMWPE MPs] = 1 g L^{-1}, [catalysts] = 1 g L^{-1} if used, [H_2O_2] = 100 mM if used, hydrothermal temperature = 140 or 160 °C and neutral pH.

After 18 h hydrothermal degradation of UHMWPE, the amount of CO_2 in the system was determined as 29.6 mg L^{-1}, and H_2 was detected as 0.4 mg L^{-1}.

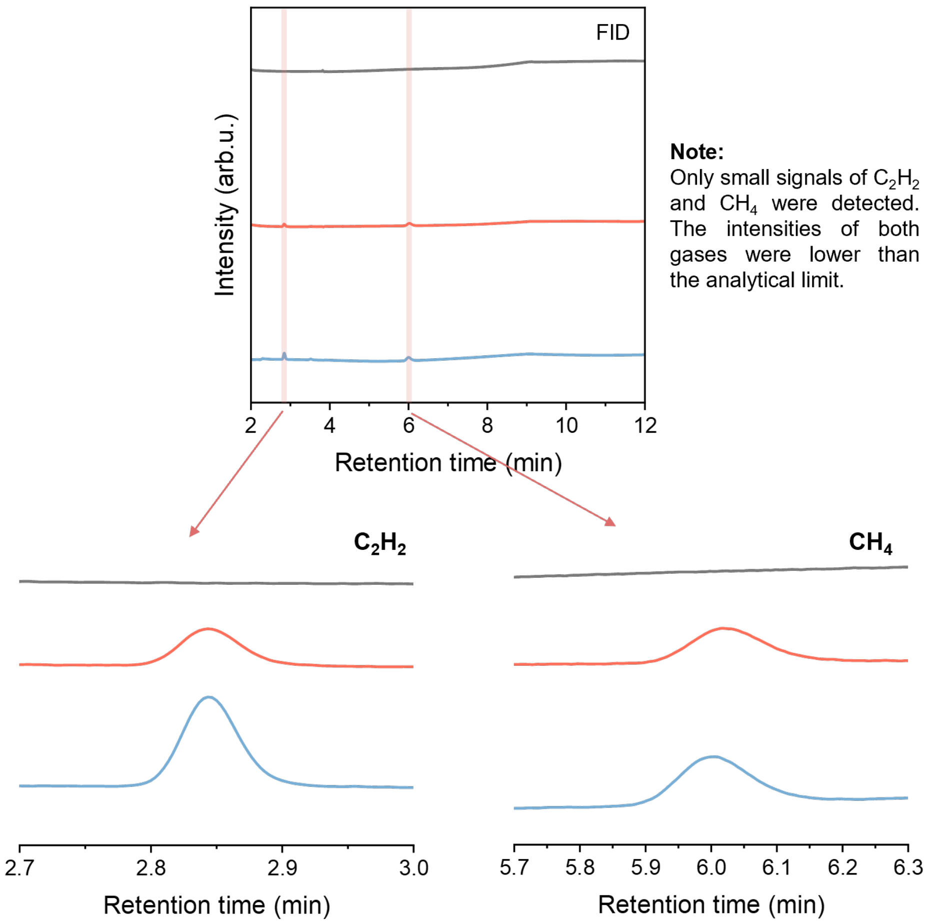

Supplementary Fig. 31 | Gas production during reaction. Detection of gases produced during hydrothermal UHMWPE MPs degradation using the 8890 GC-FID detector. Reaction conditions: reaction time = 18 h, [UHMWPE MPs] = 1 g L^{-1} if present, [catalysts] = 1 g L^{-1} if used, [H_2O_2] = 100 mM if used, hydrothermal temperature = 140 or 160 °C and neutral pH.

Only small signals of acetylene (C_2H_2) and methane (CH_4) were detected, which are below the analytical limits.

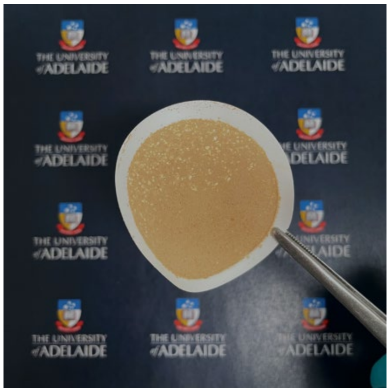

Supplementary Fig. 32 | Catalysts after reaction. FeSA-hCN catalysts collected after hydrothermal treatment. Reaction conditions: [UHMWPE MPs] = 1 g L^{-1}, [catalysts] = 1 g L^{-1}, [H_2O_2] = 100 mM, hydrothermal temperature = 140 °C and neutral pH.

During the recycling process, the catalyst was readily recovered by centrifuging to remove the unreacted MPs. After washing with water several times, the catalysts were collected by a filtration process. Fig. 4a shows the robust recyclability and chemical stability of the FeSA-hCN catalyst. The carbon nitride support processed high structural and chemical stability, even under hydrothermal conditions.

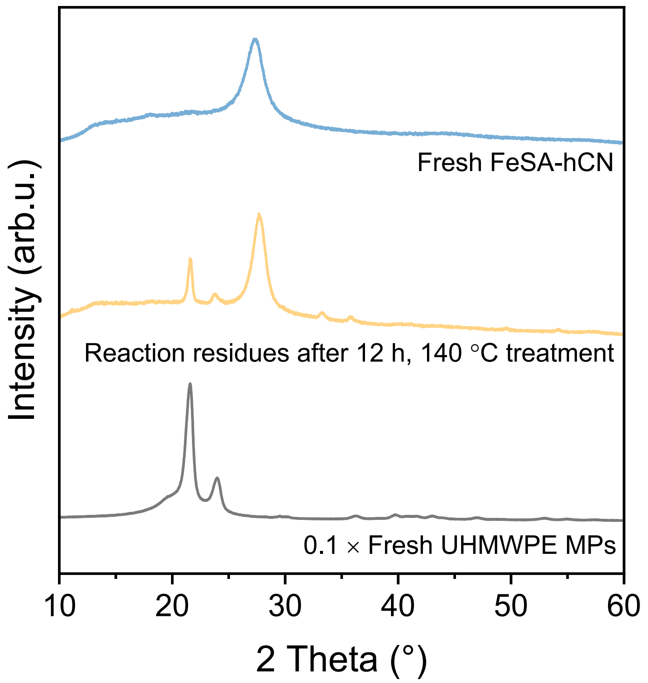

Supplementary Fig. 33 | XRD analysis of reaction residues. XRD patterns of FeSA-hCN after hydrothermal Fenton-like UHMWPE MPs degradation reaction. Reaction conditions: [UHMWPE MPs] = 1 g L^{-1}, [catalysts] = 1 g L^{-1}, [H_2O_2] = 100 mM, hydrothermal temperature = 140 °C, and neutral pH.

XRD patterns revealed that the crystal structures of FeSA-hCN remain unchanged after the hydrothermal Fenton process. No peaks associated with metallic Fe species are detected, suggesting the high dispersion of Fe species in the used FeSA-hCN. Thus, FeSA-hCN still remains the single-atom feature after harsh hydrothermal reaction conditions, confirming its good structural stability. Two characteristic peaks at around 22° and 24° can be assigned to the (110) and (200) crystal planes of UHMWPE MPs. Compared to the uncreated UHMWPE MPs, the peak intensities become lower on the reaction residues, indicating a decreased degree of crystallinity. This also confirms the successful decomposition of UHMWPE MPs.

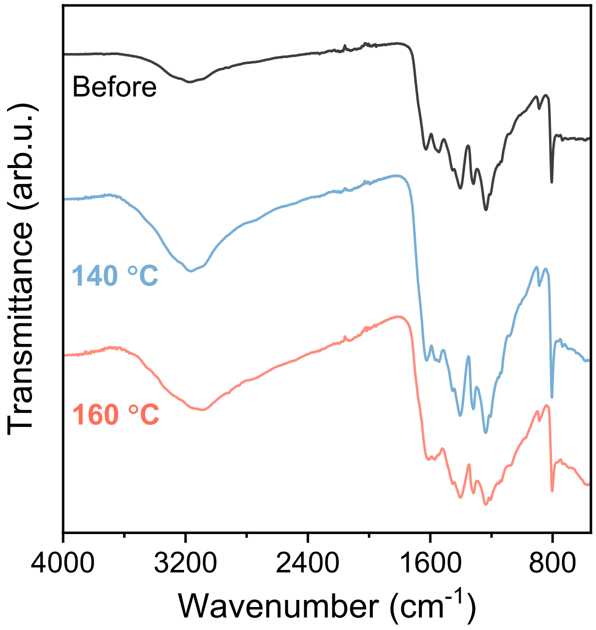

Supplementary Fig. 34 | FTIR analysis of catalysts after reaction. FTIR of used FeSA-hCN after hydrothermal Fenton-like UHMWPE MPs degradation reaction. Reaction conditions: [UHMWPE MPs] = 1 g L^{-1}, [catalysts] = 1 g L^{-1}, [H_2O_2] = 100 mM, hydrothermal temperature = 140 and 160 °C, and neutral pH.

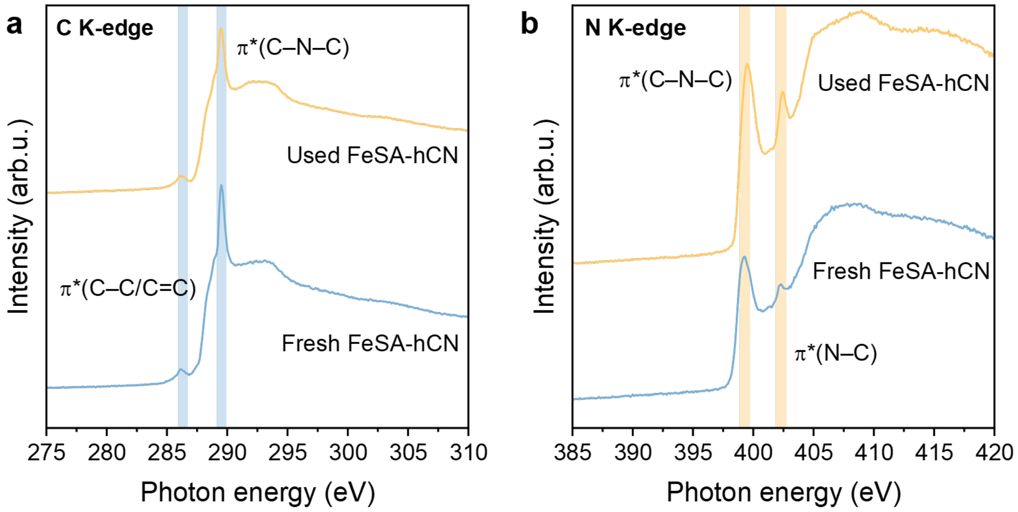

Supplementary Fig. 35 | XANES analysis of FeSA-hCN after catalytic reaction after hydrothermal Fenton-like UHMWPE MPs degradation reaction. a, C K-edge XANES. **b**, N K-edge XANES. Reaction conditions: [UHMWPE MPs] = 1 g L^{-1}, [catalysts] = 1 g L^{-1}, [H_2O_2] = 100 mM, hydrothermal temperature = 140 and 160 °C, and neutral pH.

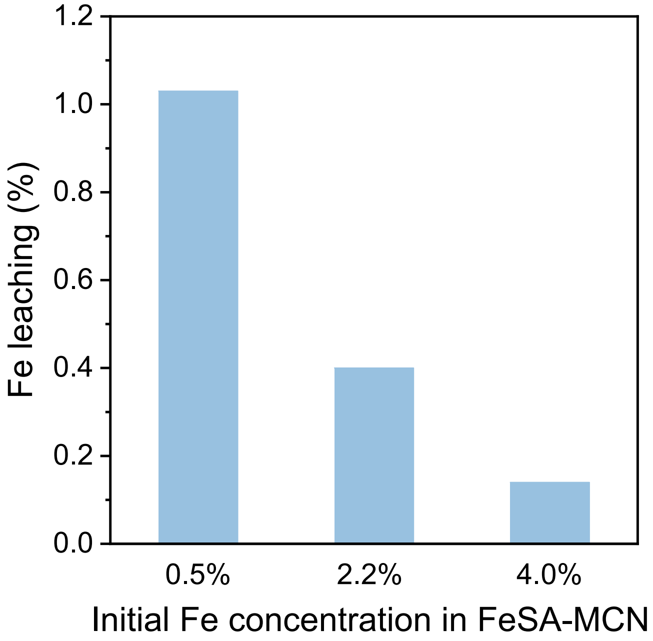

Supplementary Fig. 36 | Leaching analysis. Leaching of Fe, with respect to the initial Fe loading, into the reaction solution after hydrothermal Fenton-like UHMWPE degradation test. Reaction conditions: [UHMWPE MPs] = 1 g L^{-1}, [catalysts] = 1 g L^{-1}, [H_2O_2] = 100 mM, hydrothermal temperature = 140 °C, and neutral pH.

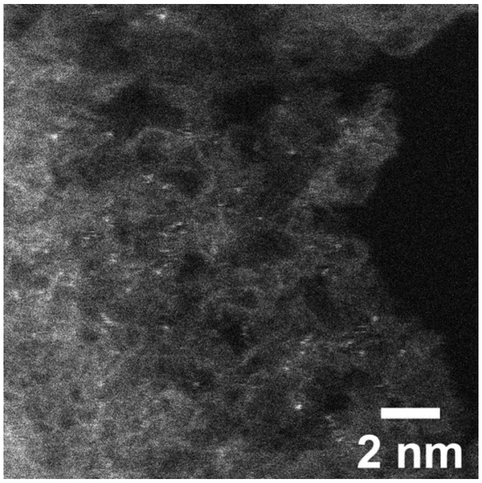

Supplementary Fig. 37 | HAADF-STEM characterization of catalysts after reaction. AC HAADF-STEM image of FeSA-hCN after hydrothermal Fenton-like UHMWPE degradation test. Fe species remained uniformly dispersed throughout the carbon nitride in the used FeSA-hCN.

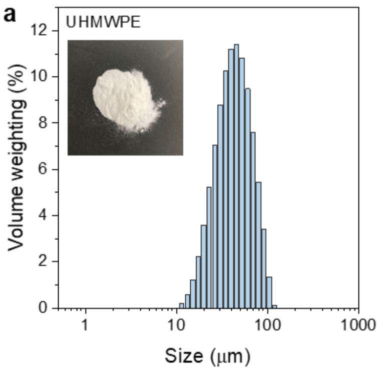

	UHMW PE	PET	HDPE	PVC	LDPE	PP	PS
D(0.1) μm	21.3	74.4	77.2	133.1	34.1	21.4	35.5
D(0.5) μm	39.6	104.0	108.0	190.2	89.3	60.2	112.3
D(0.9) μm	69.7	145.0	150.1	271.2	176.1	120.4	243.3

Notes:
D(0.1)/μm: 10% particles in the tested powders are smaller than this size.
D(0.5)/μm: 50% particles in the tested powders are smaller than this size.
D(0.9)/μm: 90% particles in the tested powders are smaller than this size.

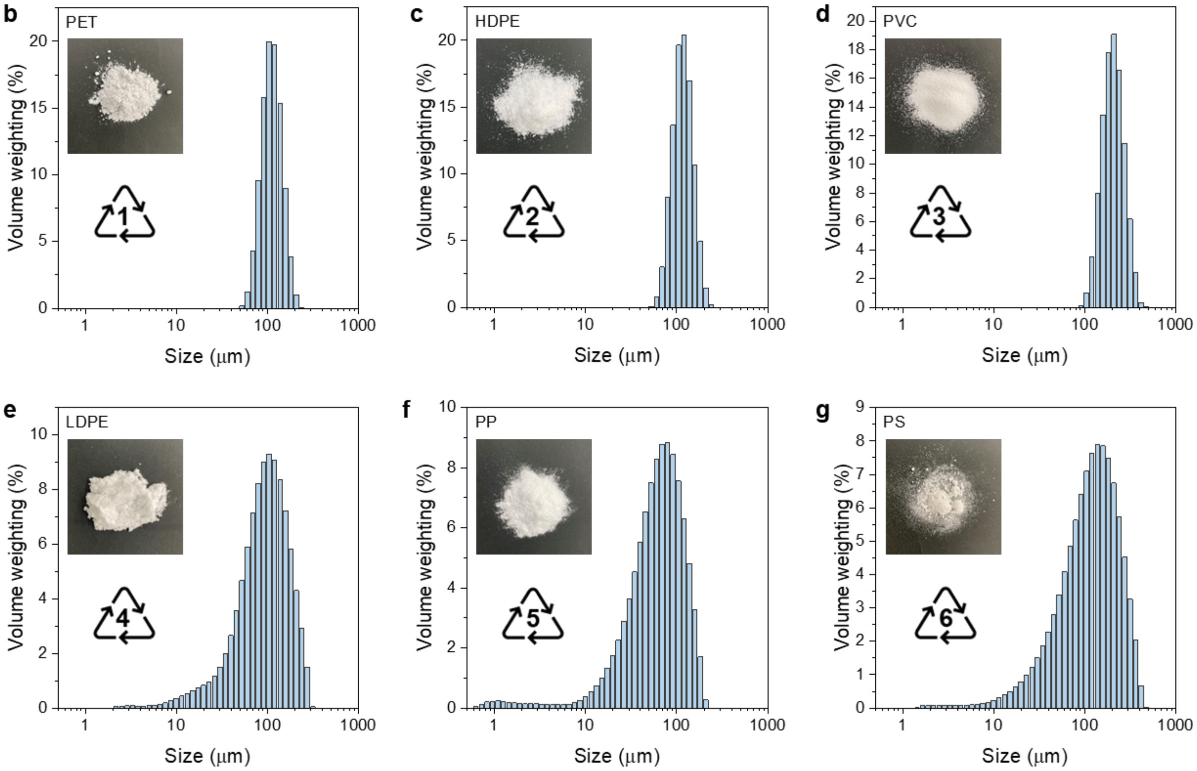

Supplementary Fig. 38 | Size analysis of microplastics. Size distribution analysis of different microplastics.

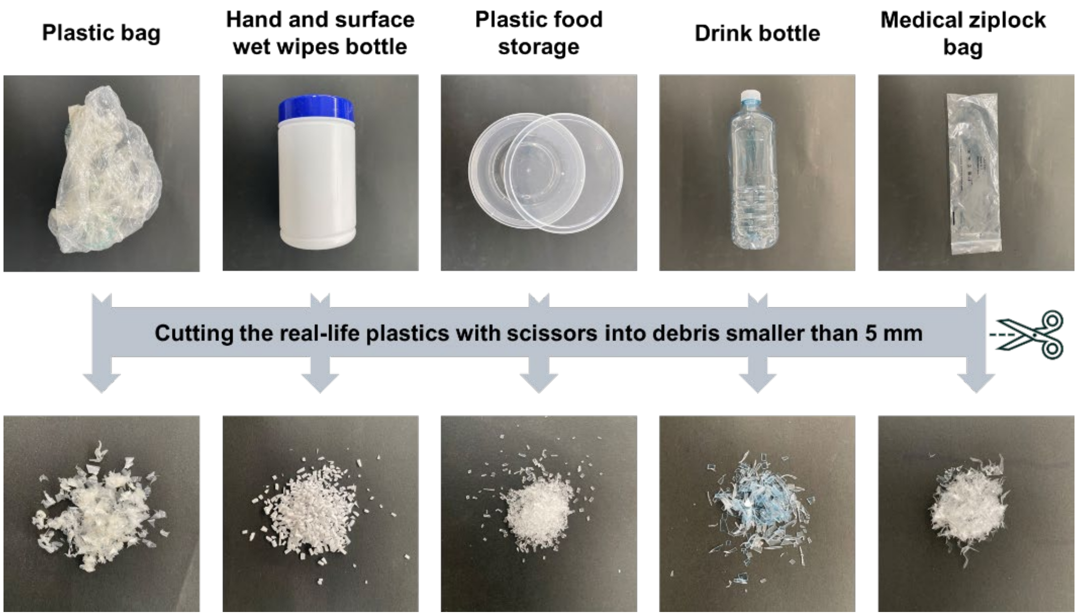

Supplementary Fig. 39 | Real-life plastics. Photos of the real-life plastics.

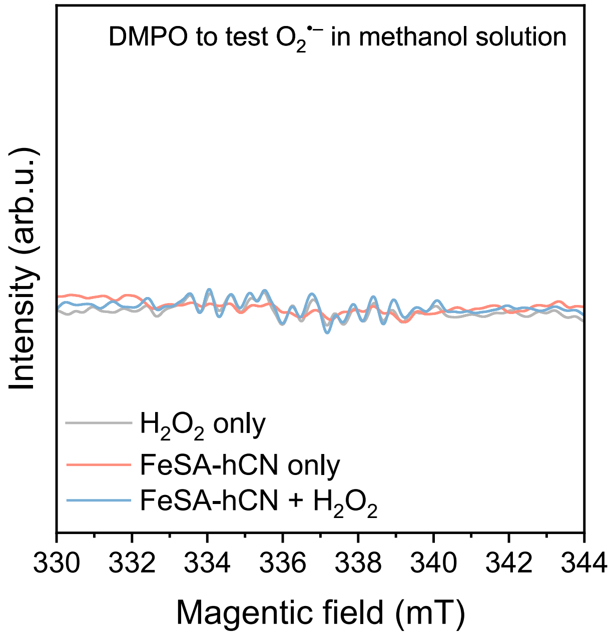

Supplementary Fig. 40 | Detection of superoxide radicals ($O_2^{\bullet-}$). DMPO spin-trapping EPR spectra of $O_2^{\bullet-}$ in a methanol/water (10% methanol) mixed solution.

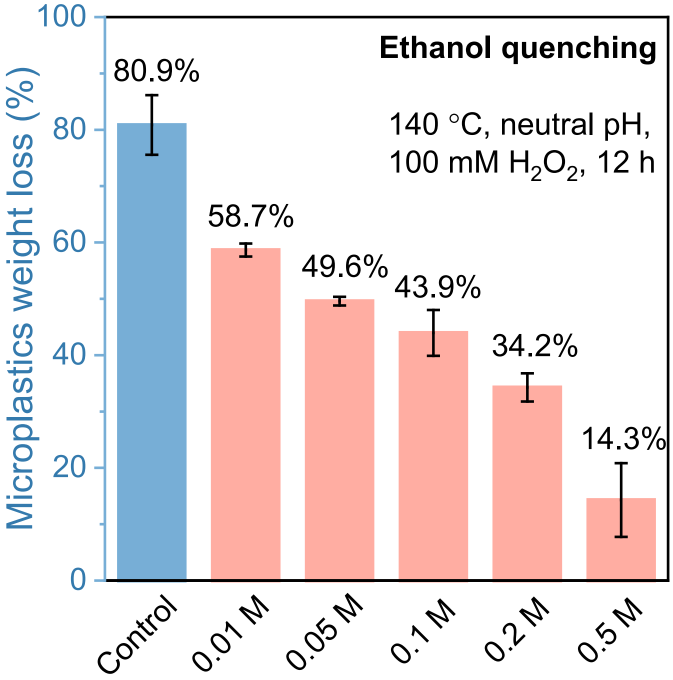

Supplementary Fig. 41 | Quenching studies for •OH radical identification. Degradation of UHMWPE in the presence of ethanol (scavenger for •OH radical).

The contribution of •OH radicals on UHMWPE degradation was studied using the radical quenching experiment with ethanol as the quenching agent (Fig. 5B and Supplementary Fig. 41). The removal efficiency was gradually reduced with increased ethanol concentration, ranging from 0.01 to 0.5 M. At the highest concentration of 0.5 M ethanol, only 14.3% of UHMWPE MPs were degraded, indicating that •OH radicals significantly contributed to the UHMWPE degradation. The error bars represent the standard deviations from triplicate tests.

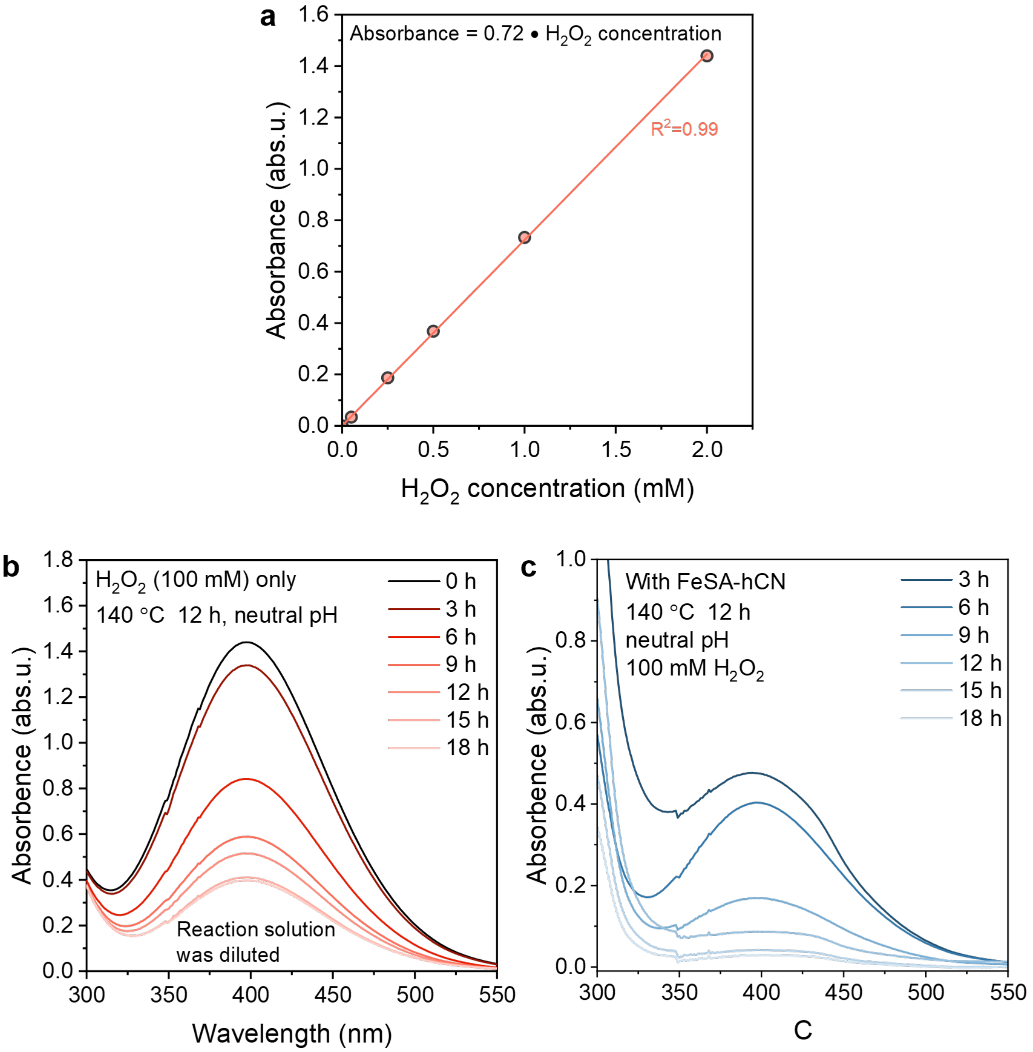

Supplementary Fig. 42 | H_2O_2 concentration in reaction solution. a, Calibration of H_2O_2 concentration. **b**, Determination of H_2O_2 concentration in reaction solution in a bare H_2O_2 hydrothermal system. **c**, Determination of H_2O_2 concentration in reaction solution in a FeSA-hCN/H_2O_2 hydrothermal system. Reaction conditions: [UHMWPE MPs] = 1 g L^{-1}, [catalysts] = 1 g L^{-1} if needed, [H_2O_2] = 100 mM, hydrothermal temperature = 140 °C, and neutral pH.

The consumption of H_2O_2 was monitored (Supplementary Fig. 42). Without catalysts, about 35.6 mM of residual H_2O_2 was detected in the solution after the reaction (Supplementary Fig. 43). In comparison, the H_2O_2 concentration dramatically decreased to about 0.7 mM in the presence of FeSA-hCN catalyst after 3 hours and decreased to 0.05 mM after an 18-hour reaction period.

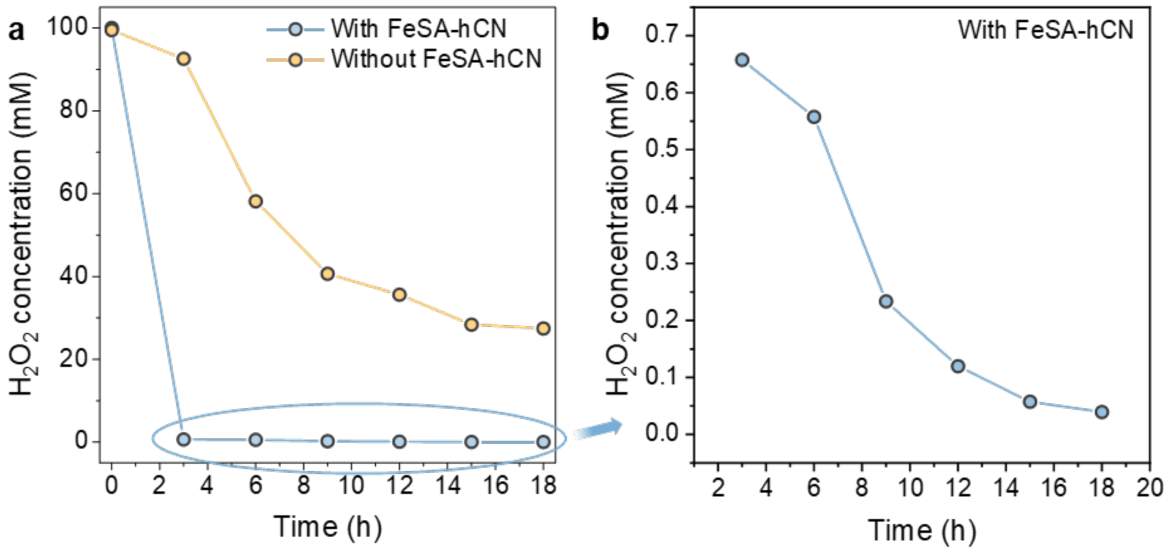

Supplementary Fig. 43 | Consumption of H_2O_2 in different systems. Reaction conditions: [UHMWPE MPs] = 1 g L^{-1}, [catalysts] = 1 g L^{-1} if needed, [H_2O_2] = 100 mM, hydrothermal temperature = 140 °C, and neutral pH.

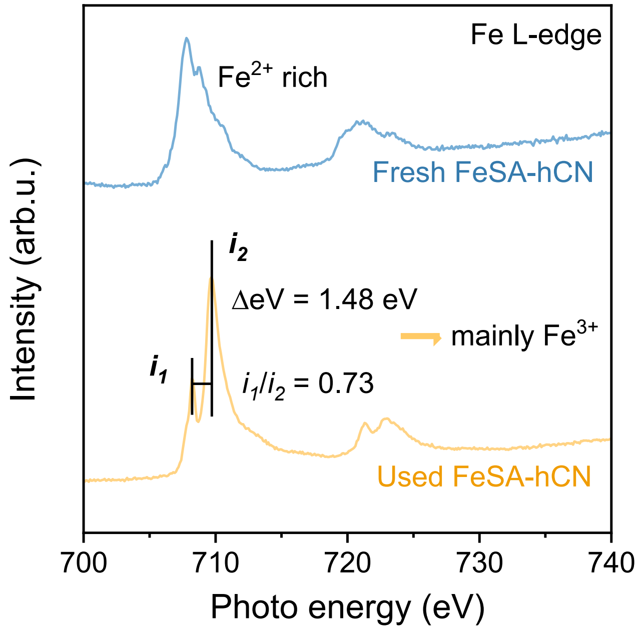

Supplementary Fig. 44 | Fe L-edge XANES analysis. Fe L-edge XANES of FeSA-hCN after hydrothermal Fenton-like UHMWPE degradation. Reaction conditions: [UHMWPE MPs] = 1 g L^{-1}, [catalysts] = 1 g L^{-1} if needed, [H_2O_2] = 100 mM, hydrothermal temperature = 140 °C, and neutral pH.

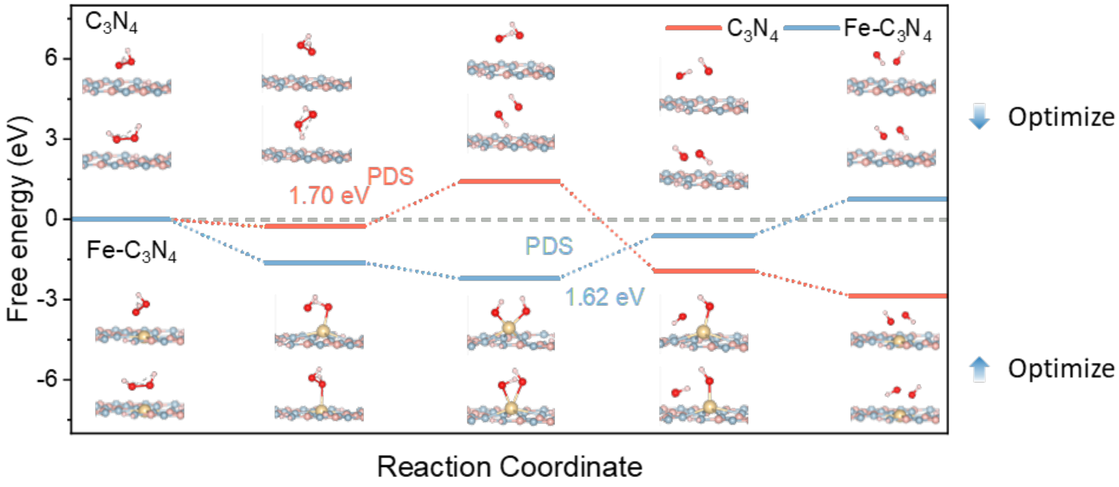

Supplementary Fig. 45 | H_2O_2 activation mechanism. Illustration of the H_2O_2 activation mechanism for •OH production. Energy profiles for the reaction process were provided. Simulated models before and after optimization were also provided. The H_2O_2 activation followed the reaction mechanism of $H_2O_2 \rightarrow {}^*H_2O_2 \rightarrow {}^*OH + {}^*OH \rightarrow OH + {}^*OH \rightarrow 2OH$.

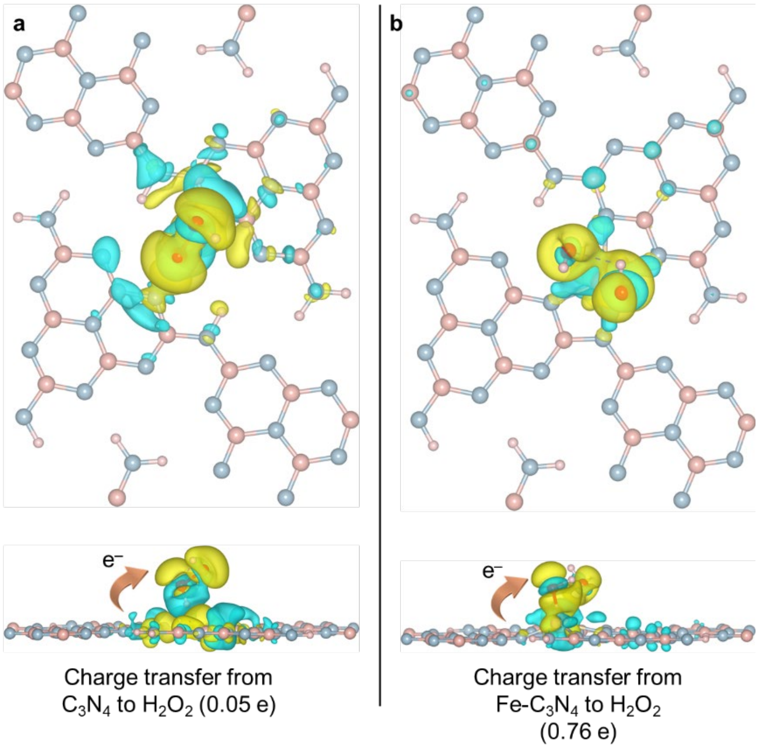

Supplementary Fig. 46 | Charge density difference with the calculated Bader charges. a, $C_3N_4{*}H_2O_2$ (charge transfer from C_3N_4 to H_2O_2 with a Bader charge of 0.05 e). **b**, Fe-$C_3N_4{*}H_2O_2$ (charge transfer from Fe-C_3N_4 to H_2O_2 with a Bader charge of 0.76 e). The yellow and cyan isosurfaces represented charge accumulation and depletion, respectively.

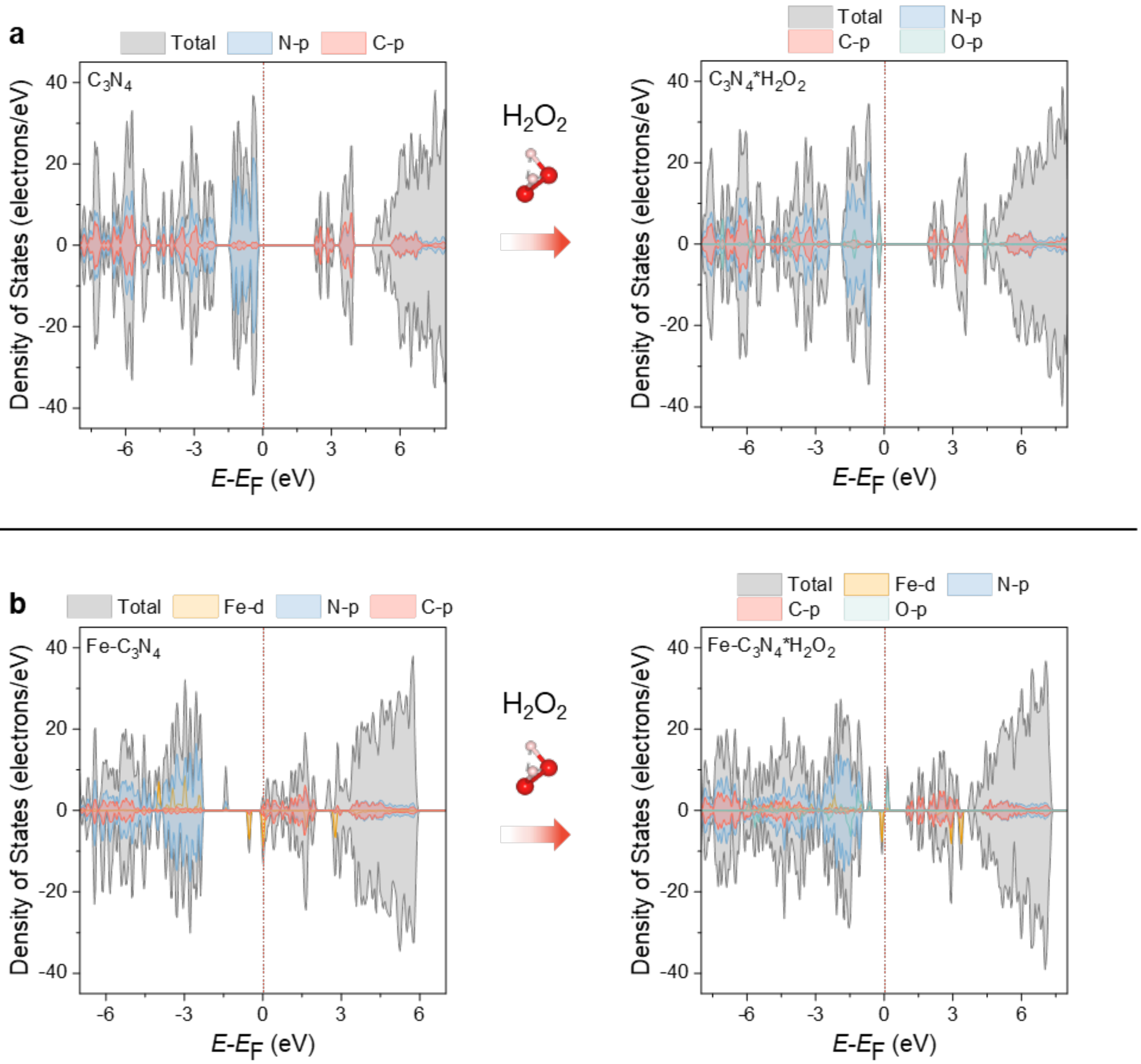

Supplementary Fig. 47 | Total and projected DOS before and after adsorbing H_2O_2. a, $C_3N_4*H_2O_2$. **b**, Fe-$C_3N_4*H_2O_2$.

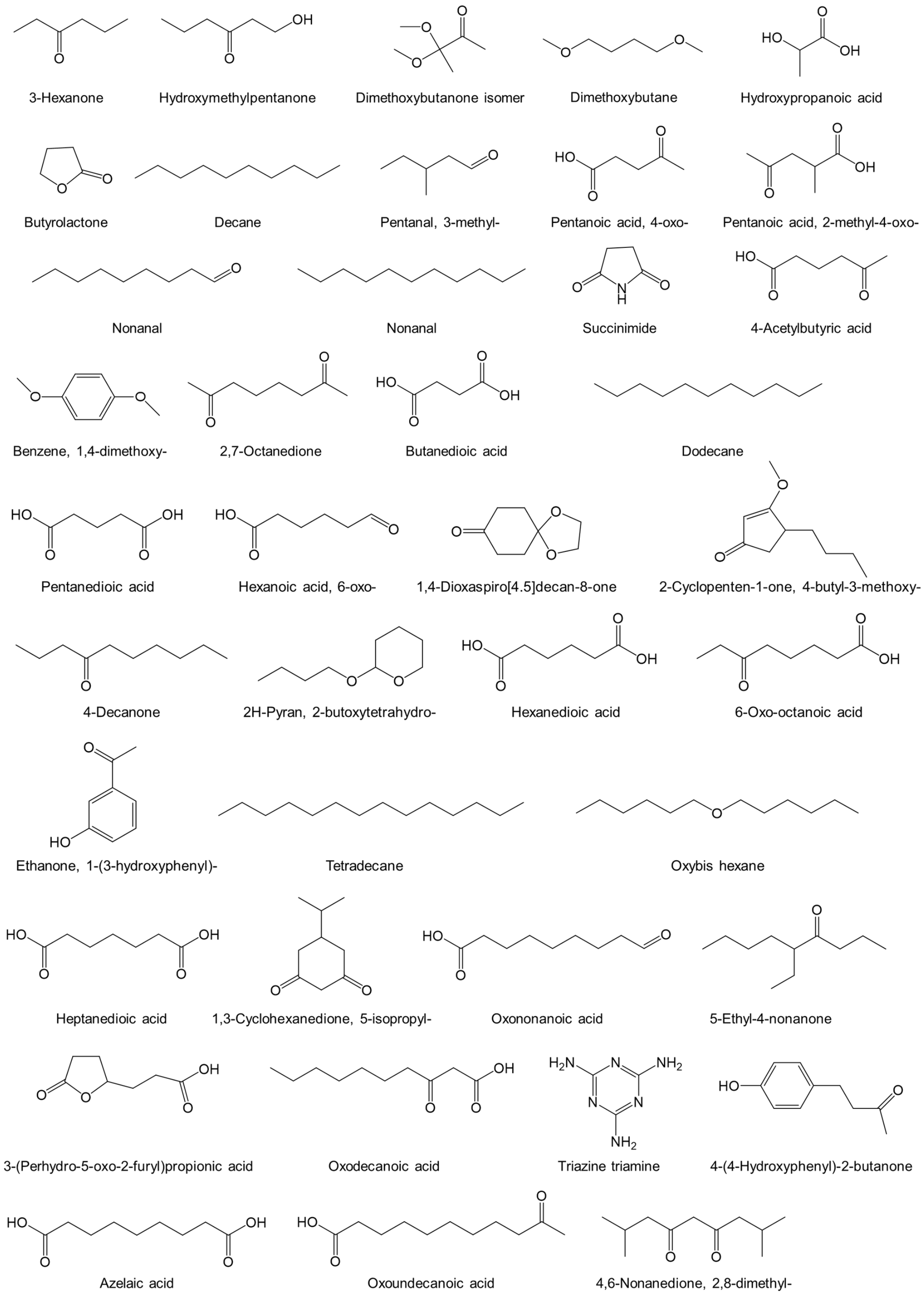

Supplementary Fig. 48 | Chemical products analysis. Chemical structure of identified products from Ret time of 3.6 to 15.3 min (Supplementary Table 7).

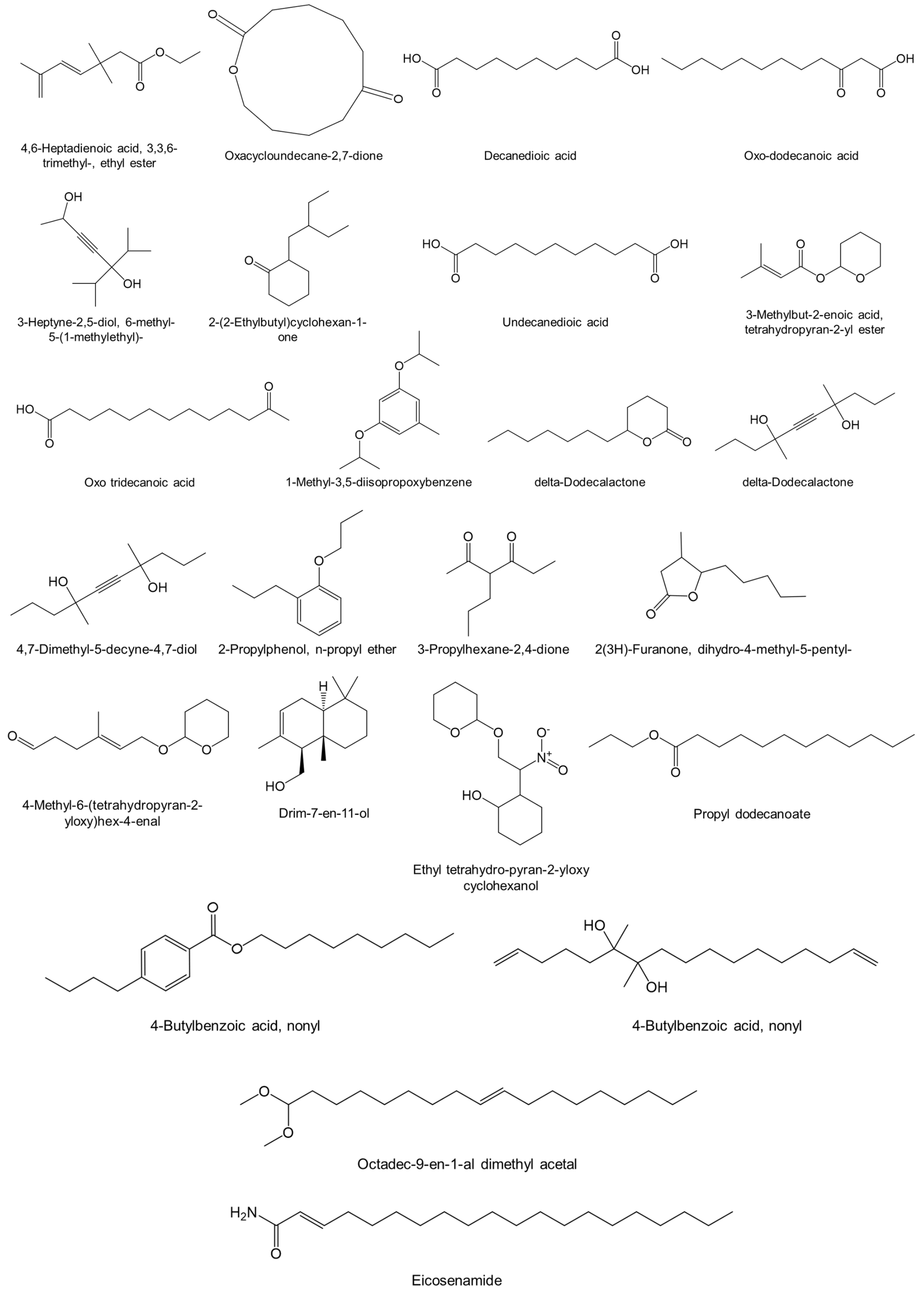

Supplementary Fig. 49 | Chemical products analysis. Chemical structure of identified products from Ret time of 15.3 to 23.8 min (Supplementary Table 7).

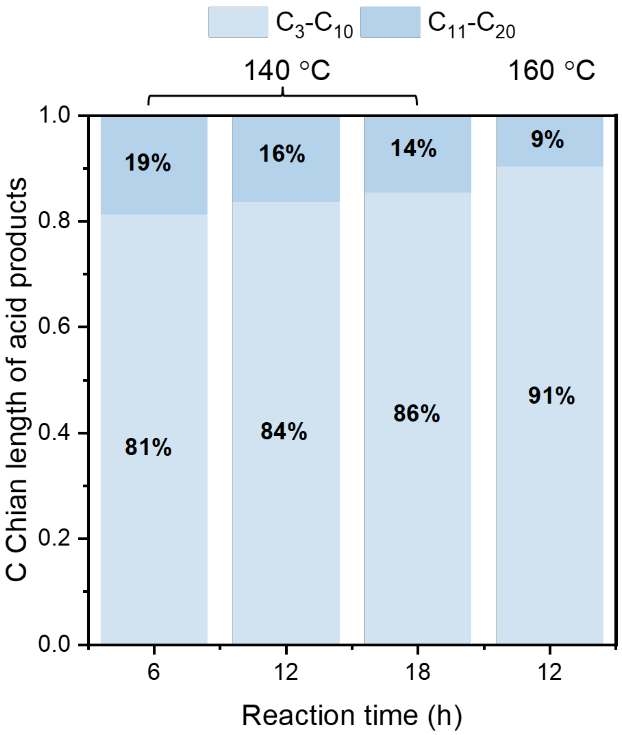

Supplementary Fig. 50 | Carbon (C) chain length analysis. C chain length of acid products after different reaction time.

The carbon chain length of the carboxylic acid products was mainly in the C_3-C_{10} range, accounting for over 80% of the carboxylic acid products. With the increased reaction time and temperature, the mass ratio of C_{11}-C_{20} fuels decreased as they were converted into shorter-chain organic products.

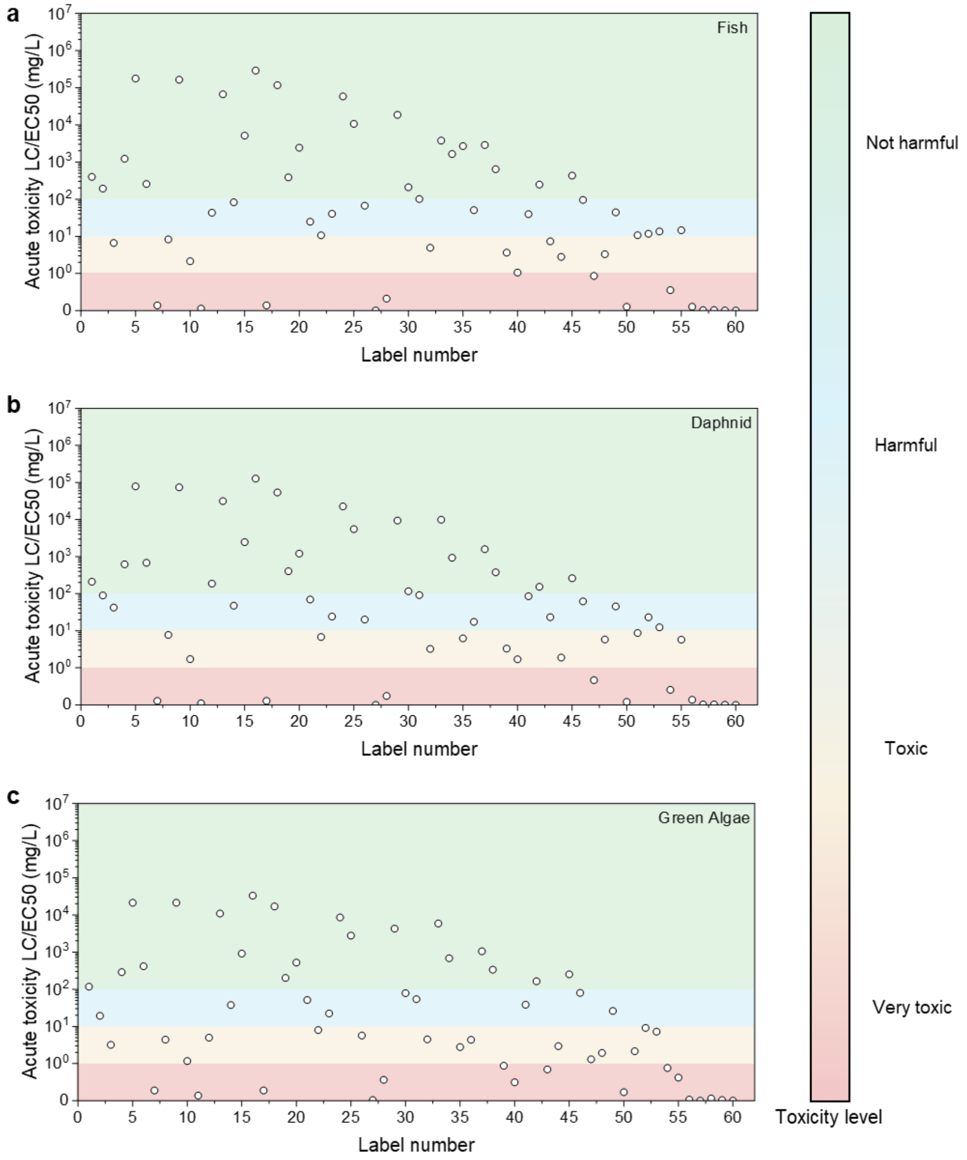

Supplementary Fig. 51 | Acute ecotoxicities of possible organic products to fish, daphnids, and green algae via Ecological Structure Activity Relationships 19 (ECOSAR) Software. a, fish. **b**, daphnids. **c**, green algae (Organic chemicals label number as shown in Supplementary Table 8).

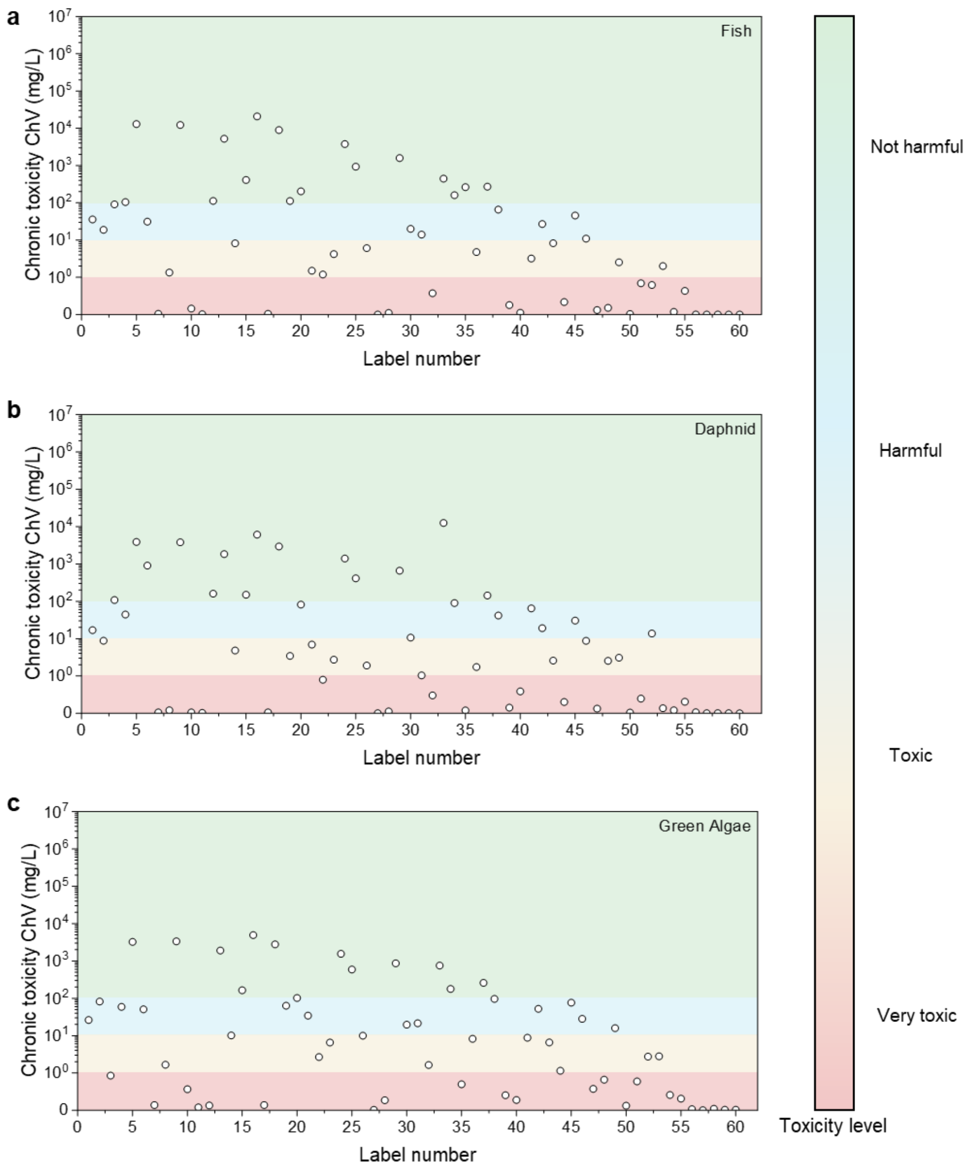

Supplementary Fig. 52 | Chronic ecotoxicities of possible organic products to fish, daphnids, and green algae via Ecological Structure Activity Relationships 19 (ECOSAR) Software. a, fish. **b**, daphnids. **c**, green algae (Organic chemicals label number as shown in Supplementary Table 8).

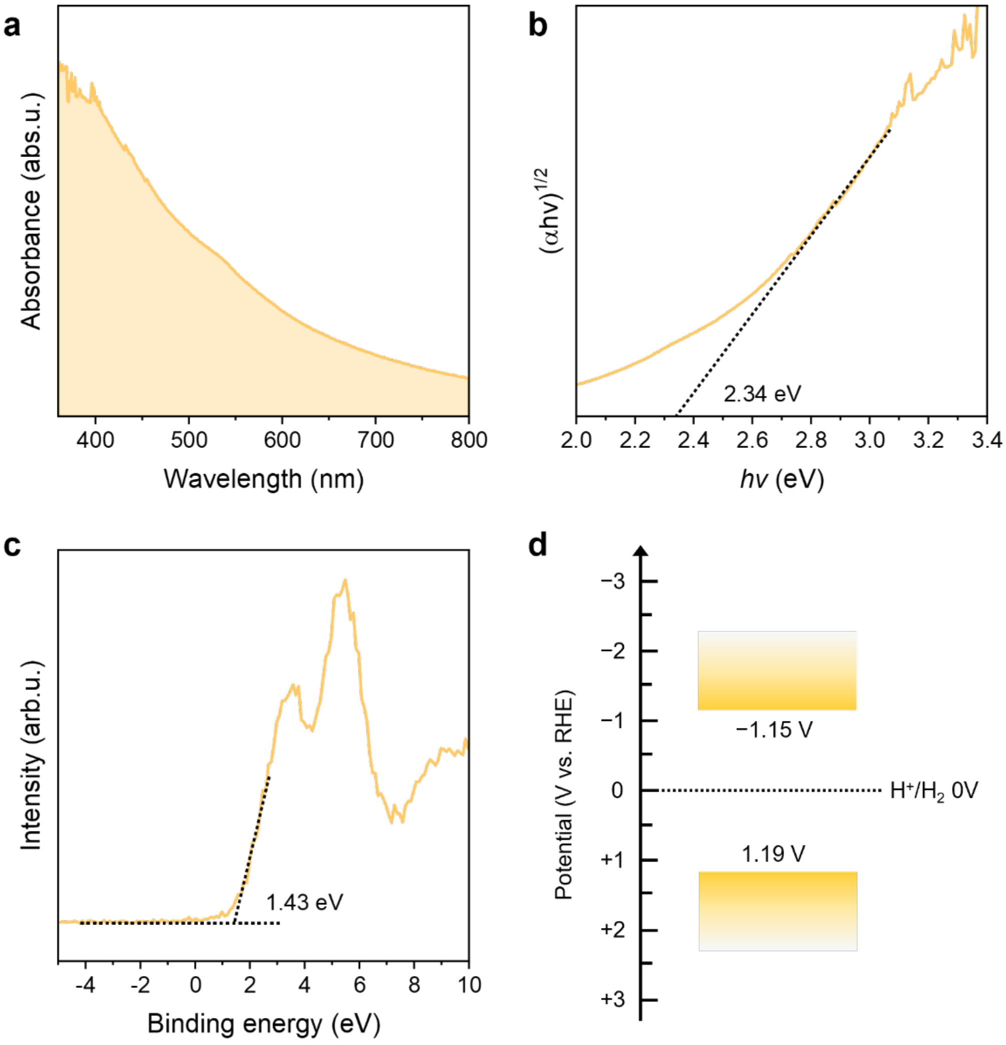

Supplementary Fig. 53 | Electronic structure analysis of catalysts after hydrothermal UHMWPE degradation. **a**, UV/Vis DRS. **b**, Tauc plot. **c**, valence band X-ray photoelectron spectroscopy (XPS). **d**, Electronic band structure.

The light absorption of the catalyst (collected after the hydrothermal UHMWPE degradation) using UV-vis diffusion reflection spectra (DRS). The bandgap energy (about 2.34 eV) was determined by the transformational Tauc plots obtained from the Kubelka-Munk function. The valence state was determined using the valence band X-ray photoelectron spectroscopy (XPS). The electronic band structure of the catalyst is shown in Supplementary Fig. 53d and Fig. 6a.

Supplementary Table 1 | Fenton/Fenton-like systems for plastics degradation.

Plastic type	Method	Catalysts	Solvent	Reaction time	Operation temperature	Plastic degradation efficiency	Product selectivity	Stability	References
					**Homogeneous system**				
PS and PE	Fenton reaction	Fe^{2+}/H_2O_2 or PDS	Water	30 days	Ambient temperature	N.M.	N.M.	N.M.	11
PET, PE. PVC, PP and EPS	Fenton reaction	Fe^{3+}/H_2O_2	Water pH=3	7.5 h	80 °C	Around 10%	N.M.	N.M.	12
UHM WPE	Hydrothermal Fenton reaction (H_2O_2)	Fe^{2+}/H_2O_2	Water pH=1	12 h	140 °C	83%	N.M.	N.M.	13
MDPE	Hydrothermal Fenton reaction (H_2O_2)	Fe^{2+}/H_2O_2	Water pH=1	16 h	140 °C	75.15%	N.M.	N.M.	14
					**Heterogeneous system**				
PE in facial cleansers	Hydrothermal Fenton reaction (PMS)	Mn@NCNTs	Water pH=3	8 h	120 °C	44%	N.M.	40 h (50% reactivity decline)	15
PVC	Electrocatalytic Fenton	TiO_2/graphite cathode	Water	6 h	100 °C	56%	N.M.	18 h run test with no catalyst powders fallen off	16
UHM WPE	Tandem hydrothermal Fenton (H_2O_2)/photocatalytic reaction	FeSA-hCN	Water neutral pH	12h	140 and 160 °C	140 °C: 81%<br>160 °C: 98%	>60% carboxylic acids	72h with no reactivity decline	This work

PS: Polystyrene
PE: Polyethylene
PET: Polyethylene Terephthalate
PVC: Polyvinyl Chloride
PP: Polypropylene
EPS: Expanded Polystyrene
HHMWPE: Ultra-High Molecular Weight Polyethylene
MDPE: Medium-Density Polyethylene
PMS: Peroxymonosulfate.
N.M.: Not Mentioned.

Supplementary Table 2 | Photocatalytic, electrocatalytic and photoelectrochemical systems for H_2 production from plastics reforming.

Plastic type	Catalytic methods	Pre-treatment methods	Catalysts	Reaction Solvent	Hydrogen production rate (from water)	References
PET	Electrocatlysis	Hydrolysis (2M KOH for 18 h at 60 °C)	Anode: CoNi0.25P<br><br>Cathode: CoNi0.25P	2M KOH	75766.7 μmol H_2 h^{-1}	17
PET	Electrocatlysis	Hydrolysis (2M KOH for 16 h at 80 °C)	Anode: B,Co-NiS<br><br>Cathode: B,Co-NiS	2M KOH	45380 μmol H_2 h^{-1}	18
PET	photovoltaic-driven electrocatalysis	Hydrolysis (1M KOH for 3 h at 180 °C, Teflon-lined autoclaves)	Anode: CuO nanowires<br><br>Cathode: Pt/C	1M KOH	1248 μmol H_2 h^{-1}	19
PET	Photoelectrocehmcial catalysis	Hydrolysis (1M NaOH for 24 h at 90 °C)	Photoanod: Ni-Pi/α-Fe_2O_3<br><br>Anode: Pt	1M NaOH	21.72 μmol H_2 cm^{-2} h^{-1}	20
PET	Photocatalysis	Hydrolysis (5M KOH for 24 h at 70 °C)	CN-CNTs-NiMo	5M KOH	0.9 μmol H_2 h^{-1}	21
PET	Photocatalysis	Hydrolysis (2M KOH for 18 h at 60 °C)	MoS_2/g-C_3N_4	2M KOH	0.05 μmol H_2 h^{-1}	22
PET	Photocatalysis	Hydrolysis (2M KOH for 18 h at 60 °C)	CPDs-CN	2M KOH	21 μmol H_2 h^{-1}	23
PET	Photocatalysis	Hydrolysis (10M KOH for 24 h at 40 °C)	CdS/CdO_x	10M NaOH	0.031 μmol H_2 h^{-1}	24
PET, PLA	Photocatalysis	Hydrolysis (2M KOH for 24 h at 40 °C)	CN_x|Ni_2P	2M KOH	PET: 0.021 μmol H_2 h^{-1}<br>PLA: 0.045 μmol H_2 h^{-1}	25
PET, PLA	Photocatalysis	Hydrolysis (2M KOH for 24 h at 60 °C)	Defect-rich $NiPS_3$	2M KOH	PET: 39.8 μmol H_2 h^{-1}<br>PLA: 31.4 μmol H_2 h^{-1}	26
UHMW PE	Photocatalysis	Hydrothermal reaction (Water/H_2O_2 at neutral pH at 140 °C)	FeSA-hCN	Water neutral pH	42 μmol H_2 h^{-1}	This work

Supplementary Table 3 | Specific area and pore volume.

	SSA, $m^2\ g^{-1}$	V_t/V_{meso}, $cm^3\ g^{-1}$
**CN**	9.6	0.06
**FeSA-hCN**	31.4	0.18

The Brunauer Emmett Teller (BET) method was used to determine the specific surface area (SSA). The total pore volume (V_t) was recorded from the amount of N_2 uptake at $P/P_0 = 0.99$. As the micropore volume is negative, Mesopore volume (V_{meso}) is the same as V_t.

Supplementary Table 4 | Energy differences (ΔeV) and intensity ratio values at Fe L_3 edge of FeSA-hCN catalysts.

	Energy differences (ΔeV)	Intensity ratio values (i_1/i_2)	Valence state of Fe
**FeSA-hCN (0.5 wt%)**	1.00	1.03	Mainly Fe^{2+}
**FeSA-hCN (2.2 wt%)**	1.01	1.03	Mainly Fe^{2+}
**Fresh FeSA-hCN (4.0 wt%)**	0.71	1.02	Mainly Fe^{2+}
**Used FeSA-hCN (4.0 wt%)**	1.48	0.73	Mainly Fe^{3+}

According to the literature[8], the valence state of Fe in FeSA-hCN catalysts were determined by their intensity ratio (absorption intensity i_1/i_2) and the energy difference between peaks i_1 and i_2.

Supplementary Table 5 | EXAFS fitting parameters at the Fe K-edge for FeSA-hCN.

Shell	C/N	R (Å)	δ^2 (10^{-3})	ΔE	R factor
Fe−N	4.1(0.3)	2.13(0.03)	9.5(0.4)	3.8(0.5)	0.02

CN: coordination numbers of identical atoms;
R: interatomic distance;
δ^2: Debye-Waller factors; ΔE: energy shift.
R factor: goodness of fit.

Error bounds that characterize the structural parameters obtained by EXAFS spectroscopy were estimated as C/N ± 20%; R ± 2%; ΔE ± 20%. EXAFS fitting results indicate that there exist Fe−N paths. The coordination number is 4.

Supplementary Table 6 | Theoretical models established for FeSA-hCN.

Model	Before optimization	After optimization	Bond length	Formation energy
1	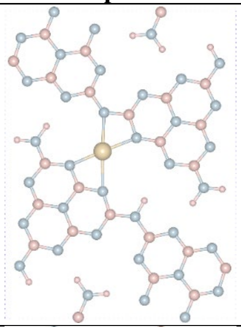	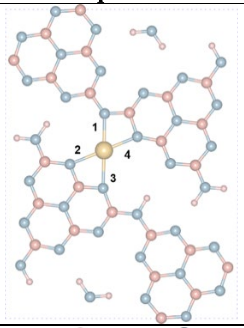	d_1=2.098 Å<br>d_2=2.069 Å<br>d_3=2.188 Å<br>d_4=2.055 Å<br>d_{ave}=2.103 Å	-3.7796 eV
2	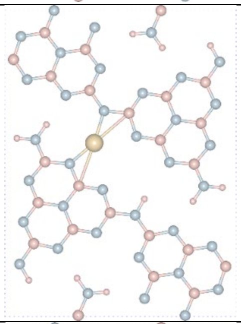	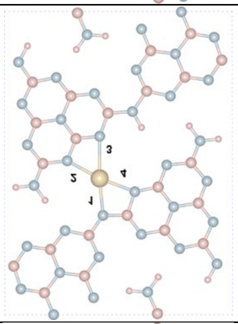	d_1=2.079 Å<br>d_2=2.069 Å<br>d_3=2.361 Å<br>d_4=2.109 Å<br>d_{ave}=2.155 Å	-3.7794 eV
3	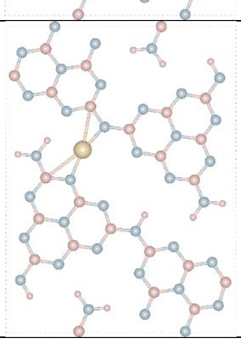	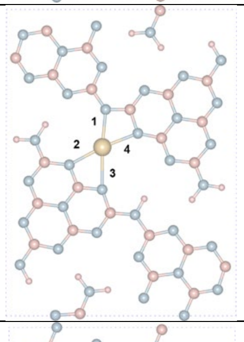	d_1=2.096 Å<br>d_2=2.083 Å<br>d_3=2.353 Å<br>d_4=2.113 Å<br>d_{ave}=2.161 Å	-3.4331 eV
4	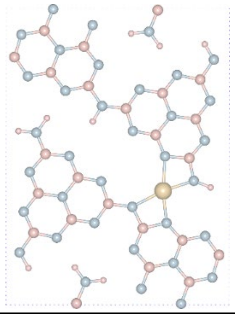	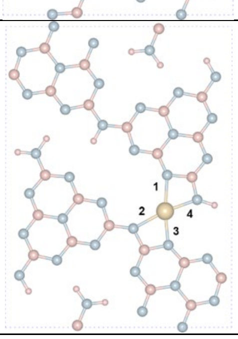	d_1=2.041 Å<br>d_2=2.048 Å<br>d_3=1.928 Å<br>d_4=1.921 Å<br>d_{ave}=1.985 Å	-2.8838 eV
Notes:<br>Fe N C H				

The thermal condensation of DCD at 550 °C promoted the formation of a hydrogen-bonded polymeric melon-based carbon nitride structure with abundant NH/NH_2 groups (Supplementary Figs. 7 and 8). This provides an ideal framework for stabilizing single-atom metals between the melon chain by bonding with surrounding C and N atoms, especially electron-abundant N sites with rich electron lone pairs. Considering the possible Fe−N(C) coordination configurations in a

melon-structured carbon nitride framework, we established four theoretical models. After the optimization using DFT calculation, it was found that Fe atoms tend to coordinate with the surrounding N atoms to form a Fe−N_4 structure. As illustrated in Supplementary Fig. 12a, there exist three types of N (N_a, N_b, N_c, Supplementary Fig. 12) in such a type of carbon nitride structure, i.e., amino groups/bridging N (sp^3-N_a), triangular edge N (sp^2-N_b), and central tertiary N (N_c). The DFT results indicated that the optimized Fe−N_4 'model 1' (by coordinating with three triangular edge N (sp^2-N_b) and one amino group/bridging N (sp^3-N_a)) had negatively lower formation energy than the others, and therefore, is thermodynamically favorable to exist.

Supplementary Table 7 | Hydrothermal oxidation intermediates identified by GC-MS using NIST14 spectral library as the database.

Ret Time	Sample identification	Molecular formula	Molecular mass, g mol^{-1}
3.6	3-Hexanone	$C_6H_{12}O$	100.16
3.9	Hydroxymethylpentanone	$C_6H_{12}O_2$	116.16
3.9	Dimethoxybutanone isomer	$C_6H_{12}O_3$	132.16
4	Dimethoxybutane	$C_6H_{14}O_2$	118.17
4.8	Hydroxypropanoic acid	$C_3H_6O_3$	90.08
4.9	Butyrolactone	$C_4H_6O_2$	86.09
6.2	Decane	$C_{10}H_{22}$	142.28
6.4	Pentanal, 3-methyl-	$C_6H_{12}O$	100.16
7	Pentanoic acid, 4-oxo-	$C_5H_8O_3$	116.12
7.7	Pentanoic acid, 2-methyl-4-oxo-	$C_6H_{10}O_3$	130.14
7.8	Nonanal	$C_9H_{18}O$	142.24
7.9	Undecane	$C_{11}H_{24}$	156.31
8.2	Succinimide	$C_4H_5NO_2$	99.09
8.4	4-Acetylbutyric acid	$C_6H_{10}O_3$	130.14
8.7	Benzene, 1,4-dimethoxy-	$C_8H_{10}O_2$	138.16
8.8	2,7-Octanedione	$C_8H_{14}O_2$	142.2
8.9	Butanedioic acid	C4H6O4	118.09
9.3	Dodecane	$C_{12}H_{26}$	170.33
9.8	Pentanedioic acid	$C_5H_8O_4$	132.11
10	Hexanoic acid, 6-oxo-	$C_6H_{10}O_3$	130.14
10.5	1,4-Dioxaspiro[4.5]decan-8-one	$C_8H_{12}O_3$	156.18
10.6	2-Cyclopenten-1-one, 4-butyl-3-methoxy-	$C_{10}H_{16}O_2$	168.23
10.9	4-Decanone	$C_{10}H_{20}O$	156.27
10.9	2H-Pyran, 2-butoxytetrahydro-	$C_9H_{18}O$	142.24
11.1	Hexanedioic acid	$C_6H_{10}O_4$	146.14
11.4	6 Oxo octanoic acid	$C_8H_{14}O_3$	158.09
11.9	Ethanone, 1-(3-hydroxyphenyl)-	$C_8H_8O_2$	136.15
12	Tetradecane	$C_{14}H_{30}$	198.39

12.2	Piperazine homologue	-	-
12.3	Oxybis hexane	$C_{12}H_{26}O$	186.33
12.4	Heptanedioic acid	$C_7H_{12}O_4$	160.17
12.6	1,3-Cyclohexanedione, 5-isopropyl-	$C_{10}H_{16}O_2$	168.23
12.6	Furanone homologue	-	-
12.7	Oxononanoic acid	$C_9H_{16}O_3$	172.22
13.1	5-Ethyl-4-nonanone	$C_{11}H_{22}O$	170.29
13.5	3-(Perhydro-5-oxo-2-furyl)propionic acid	$C_9H_{12}O_4$	184.19
13.8	Cyclohexanone homologue	-	-
13.9	Oxodecanoic acid	$C_{10}H_{18}O_3$	186.25
14.3	Triazine triamine	$C_3H_6N_6$	126.12
14.6	4-(4-Hydroxyphenyl)-2-butanone	$C_{10}H_{12}O_2$	164.20
14.7	Azelaic acid	$C_9H_{16}O_4$	188.22
14.9	Furanone isomer	-	-
15.1	Oxoundecanoic acid	$C_{11}H_{20}O_3$	200.27
15.3	Decenone isomer	-	-
15.3	4,6-Nonanedione, 2,8-dimethyl-	$C_{11}H_{20}O_2$	184.28
15.6	4,6-Heptadienoic acid, 3,3,6-trimethyl-, ethyl ester	$C_{13}H_{22}O_2$	210.31
15.7	Oxacycloundecane-2,7-dione	$C_{11}H_{18}O_3$	198.26
15.8	Decanedioic acid	$C_{10}H_{18}O_4$	202.25
16.1	Furanone homologue	-	-
16.2	Oxo-dodecanoic acid	$C_{12}H_{24}O_3$	216.32
16.4	Dimethyl-2-methoxy-6-nitro-Benzenacetic amide	$C_{10}H_{12}N_2O_4$	224.21
16.5	3-Heptyne-2,5-diol, 6-methyl-5-(1-methylethyl)-	$C_{10}H_{18}O_2$	170.25
16.6	2-(2-Ethylbutyl)cyclohexan-1-one	$C_{12}H_{22}O$	182.30
16.7	Undecanedioic acid	$C_{11}H_{20}O_4$	216.27
17	Furanone homologue	-	-
17.2	Oxo tridecanoic acid	$C_{13}H_{24}O_3$	228.33
17.5	1-Methyl-3,5-diisopropoxybenzene	$C_{13}H_{20}O_2$	208.3
17.6	Delta-Dodecalactone	$C_{12}H_{22}O_2$	198.3
17.6	4,7-Dimethyl-5-decyne-4,7-diol	$C_{12}H_{22}O_2$	198.3
17.9	2-Propylphenol, n-propyl ether	$C_{12}H_{18}O$	178.27

18.3	Furanone homologue	-	-
18.5	3-Propylhexane-2,4-dione	$C_9H_{16}O_2$	156.22
18.5	2(3H)-Furanone, dihydro-4-methyl-5-pentyl-	$C_{10}H_{18}O_2$	170.25
18.7	4-Methyl-6-(tetrahydropyran-2-yloxy)hex-4-enal	$C_{12}H_{20}O_3$	212.29
18.9	Drim-7-en-11-ol	$C_{15}H_{26}O$	222.37
19.3	Furanone homologue	-	-
19.7	Ethyl tetrahydro-pyran-2-yloxy cyclohexanol	$C_{13}H_{23}NO_5$	273.33
20.2	bis Propyl-Dodecalactone	$C_{15}H_{30}O_2$	242.40
20.7	4-Butylbenzoic acid, nonyl	$C_{20}H_{32}O$	288.47
22	Dimethyl-Octadecadiene-diol	$C_{20}H_{38}O_2$	310.53
22.6	Octadec-9-en-1-al dimethyl acetal	$C_{20}H_{40}O_2$	312.5
23.6	Eicosenamide isomer	$C_{20}H_{39}NO$	309.53
23.7	Eicosanamide isomer	$C_{20}H_{39}NO$	309.53
23.8	Eicosanamide isomer	$C_{20}H_{39}NO$	309.53

Supplementary Table 8 | Label number of chemicals in ecotoxicities (including acute and chronic toxicities) analysis for Supplementary Figs. 48 and 49.

Label number	Chemical name
1	3-Hexanone
2	Hydroxymethylpentanone
3	3,3-Dimethoxybutan-2-one
4	Dimethoxybutane
5	Hydroxypropanoic acid
6	Butyrolactone
7	Decane
8	Pentanal, 3-methyl-
9	Pentanoic acid, 4-oxo-
10	Nonanal
11	Undecane
12	Succinimide
13	4-Acetylbutyric acid
14	Benzene, 1,4-dimethoxy-
15	2,7-Octanedione
16	Butanedioic acid
17	Dodecane
18	pentanedioic acid
19	Hexanoic acid, 6-oxo-
20	1,4-Dioxaspiro[4.5]decan-8-one
21	2-Cyclopenten-1-one, 4-butyl-3-methoxy-
22	4-Decanone
23	2H-Pyran, 2-butoxytetrahydro-
24	Hexanedioic acid
25	6-Oxo-octanoic acid
26	Ethanone, 1-(3-hydroxyphenyl)-
27	Tetradecane

28	Oxybis hexane
29	Heptanedioic acid
30	1,3-Cyclohexanedione, 5-isopropyl-
31	Oxononanoic acid
32	5-Ethyl-4-nonanone
33	3-(Perhydro-5-oxo-2-furyl)propionic acid
34	Oxodecanoic acid
35	Triazine triamine
36	4-(4-Hydroxyphenyl)-2-butanone
37	Azelaic acid
38	Oxoundecanoic acid
39	4,6-Nonanedione, 2,8-dimethyl-
40	4,6-Heptadienoic acid, 3,3,6-trimethyl-, ethyl ester
41	Oxacycloundecane-2,7-dione
42	Oxo-dodecanoic acid
43	3-Heptyne-2,5-diol, 6-methyl-5-(1-methylethyl)-
44	2-(2-Ethylbutyl)cyclohexan-1-one
45	Undecanedioic acid
46	3-Methylbut-2-enoic acid, tetrahydropyran-2-yl ester
47	1-Methyl-3,5-diisopropoxybenzene
48	delta-Dodecalactone
49	4,7-Dimethyl-5-decyne-4,7-diol
50	2-Propylphenol, n-propyl ether
51	3-Propylhexane-2,4-dione
52	2(3H)-Furanone, dihydro-4-methyl-5-pentyl-
53	4-Methyl-6-(tetrahydropyran-2-yloxy)hex-4-enal
54	Drim-7-en-11-ol
55	Ethyl tetrahydro-pyran-2-yloxy cyclohexanol
56	Propyl dodecanoate
57	4-Butylbenzoic acid, nonyl

58	9-Octadecene, 1,1-dimethoxy-
59	Octadec-9-en-1-al dimethyl acetal
60	Eicosenamide

Supplementary References

1. Stöber, W., Fink, A. & Bohn, E. Controlled growth of monodisperse silica spheres in the micron size range. *J. Colloid Interface Sci.* **26**, 62–69 (1968).
2. Lin, J. *et al.* Macroporous carbon-nitride-supported transition-metal single-atom catalysts for photocatalytic hydrogen production from ammonia splitting. *ACS Catal.* **13**, 11711–11722 (2023).
3. Lei, J. *et al.* Robust photocatalytic H_2O_2 production over inverse opal g-C_3N_4 with carbon vacancy under visible light. *ACS Sustain. Chem. Eng.* **7**, 16467–16473 (2019).
4. Tan, H. *et al.* Photocatalysis of water into hydrogen peroxide over an atomic Ga-N_5 site. *Nat. Synth.* **2**, 557–563 (2023).
5. Cheng, L., Yin, H., Cai, C., Fan, J. & Xiang, Q. Single Ni atoms anchored on porous few-layer g-C_3N_4 for photocatalytic CO_2 reduction: The role of edge confinement. *Small* **16**, 2002411 (2020).
6. Niu, P., Zhang, L., Liu, G. & Cheng, H. M. Graphene-like carbon nitride nanosheets for improved photocatalytic activities. *Adv. Funct. Mater.* **22**, 4763–4770 (2012).
7. Hocking, R. K. *et al.* Fe L-Edge X-ray absorption spectroscopy determination of differential orbital covalency of siderophore model compounds: electronic structure contributions to high stability constants. *J. Am. Chem. Soc.* **132**, 4006–4015 (2010).
8. von der Heyden, B. P., Roychoudhury, A. N., Mtshali, T. N., Tyliszczak, T. & Myneni, S. C. B. Chemically and geographically distinct solid-phase iron pools in the southern ccean. *Science* **338**, 1199–1201 (2012).
9. Jin, H. *et al.* Regulating the electronic structure through charge redistribution in dense single-atom catalysts for enhanced alkene epoxidation. *Nat. Commun.* **14**, 1–10 (2023).
10. Peng, P. *et al.* A pyrolysis-free path toward superiorly catalytic nitrogen-coordinated single atom. *Sci. Adv.* **5**, 1–8 (2019).
11. Liu, P. *et al.* New insights into the aging behavior of microplastics accelerated by advanced oxidation processes. *Environ. Sci. Technol.* **53**, 3579–3588 (2019).
12. Ortiz, D. *et al.* Insights into the degradation of microplastics by Fenton oxidation: From surface modification to mineralization. *Chemosphere* **309**, 136809 (2022).
13. Hu, K. *et al.* Degradation of microplastics by a thermal Fenton reaction. *ACS ES&T Engg.* **2**, 110–120 (2022).
14. Singh, V., Park, S. Y. & Kim, C. G. Hybrid oxidation of microplastics with Fenton and hydrothermal reactions. *ACS EST Water* (2024) doi:10.1021/acsestwater.3c00742.
15. Kang, J. *et al.* Degradation of cosmetic microplastics via functionalized carbon nanosprings. *Matter* **1**, 745–758 (2019).
16. Miao, F. *et al.* Degradation of polyvinyl chloride microplastics via an electro-Fenton-like system with a TiO_2/graphite cathode. *J. Hazard. Mater.* **399**, 123023 (2020).
17. Zhou, H. *et al.* Electrocatalytic upcycling of polyethylene terephthalate to commodity chemicals and H_2 fuel. *Nat. Commun.* **12**, 1–9 (2021).
18. Chen, Z., Wei, W., Shen, Y. & Ni, B. J. Defective nickel sulfide hierarchical structures for efficient electrochemical conversion of plastic waste to value-added chemicals and hydrogen fuel. *Green Chem.* **25**, 5979–5988 (2023).
19. Zhang, T. *et al.* Photovoltaic-driven electrocatalytic upcycling poly(ethylene terephthalate) plastic waste coupled with hydrogen generation. *J. Hazard. Mater.* **450**, 131054 (2023).
20. Zhang, B. *et al.* Photoelectrochemical conversion of plastic waste into high-value chemicals

coupling hydrogen production. *Chem. Eng. J.* **462**, 142247 (2023).

21. Gong, X. *et al.* Photoreforming of plastic waste poly (ethylene terephthalate) via in-situ derived CN-CNTs-NiMo hybrids. *Appl. Catal. B Environ.* **307**, 121143 (2022).
22. Kang, H. *et al.* Concentration-dependent photocatalytic upcycling of poly(ethylene terephthalate) plastic waste. *ACS Mater. Lett.* **5**, 3032–3041 (2023).
23. Han, M., Zhu, S., Xia, C. & Yang, B. Photocatalytic upcycling of poly(ethylene terephthalate) plastic to high-value chemicals. *Appl. Catal. B Environ.* **316**, 121662 (2022).
24. Uekert, T., Kuehnel, M. F., Wakerley, D. W. & Reisner, E. Plastic waste as a feedstock for solar-driven H_2 generation. *Energy Environ. Sci.* **11**, 2853–2857 (2018).
25. Uekert, T., Kasap, H. & Reisner, E. Photoreforming of nonrecyclable plastic waste over a carbon nitride/nickel phosphide catalyst. *J. Am. Chem. Soc.* **141**, 15201–15210 (2019).
26. Zhang, S. *et al.* Boosted photoreforming of plastic waste via defect-rich $NiPS_3$ nanosheets. *J. Am. Chem. Soc.* **145**, 6410–6419 (2023).
